# Miniaturized equal/unequal Wilkinson power dividers capable of harmonic suppression utilizing microstrip π-shaped resonators modified by lumped elements

**DOI:** 10.1038/s41598-024-57940-z

**Published:** 2024-03-27

**Authors:** Ashkan Abdipour, Seyed Vahab Al-Din Makki

**Affiliations:** Electrical Engineering Department, Faculty of Engineering, Razi University, Kermanshah, Iran

**Keywords:** Microstrip Wilkinson power divider, Modified π-shaped resonator, Lumped elements, Selectable operating frequency and power division ratio, Equal power division, Unequal power division, Engineering, Electrical and electronic engineering, Electronics, photonics and device physics

## Abstract

In this paper, modified π-shaped resonator composed of both microstrip transmission lines and lumped elements are employed to design a Wilkinson power divider. Utilizing these resonators leads to designing a compact divider featuring a selectable operating frequency with optional power division ratio and very wide-range harmonic suppression. To vary the operating frequency and the power division ratio, the values of just the utilized lumped elements are changed without manipulating the dimensions of microstrip lines. As a design sample, a miniaturized divider capable of operating at four frequencies i.e., 0.5, 1.0, 1.5 and 2 GHz with optional equal or unequal power division and harmonic suppression ability at each of these frequencies is designed and simulated. Finally, as a feasible sample, another Wilkinson power divider which can optionally operate at 700 MHz with equal power division or 1.2 GHz with unequal power division is designed and implemented. Based on the measurement results, the spurious harmonics from 2nd to 25th in the 700 MHz-divider and 2nd to 15th in the 1.2 GHz-divider are suppressed. Moreover, almost 96% and 93% size reduction at 700 MHz and 1.2 GHz, respectively, are achieved. The S21 and S31of the unequal divider are − 8.8 and − 3.73 dB, which indicate an unequal 3.2:1 power division.

## Introduction

Since the introduction of the first power divider (PD) by Wilkinson^1^, the communication technology has experienced an evolutionary period. Thus, redesigning the conventional power divider to improve its performance can be helpful. To promote the characteristics of the conventional PD, various designs have been proposed so far. In^2^, applying transmission line segments to two transformers results in suppressing the nth-order harmonics, but using this technique enhances the occupied area of this PD. In^3^, by using parallel coupled lines with one end connected in series with an open stub a PD with above 37 dB rejection for the second, third and fourth spurious harmonics and almost 20% miniaturization have been presented. To achieve a PD with better slow-wave factor in^4^, crossing bond wires have been employed which have resulted in better than 40 dB rejection for the third and fifth harmonics and 50% size reduction. To omit high-order harmonics via slow-wave and band-stop characteristics, various effective techniques such as the electromagnetic bandgap (EBG)^5,6^ and the defected ground structure (DGS)^7,8^ have been utilized to design Wilkinson PDs. In some samples, Wilkinson PDs are designed to either omit spurious harmonics^9^, or decrease the occupied area^10^. In^11^, to reduce the circuit size and reject spurious harmonics, slow-wave loading has been employed and second to fifth unwanted harmonics have been rejected. In^12^, a wideband bandpass PD adopting a simple ring resonator has been presented. As another technique, open-stub transmission lines have been used to propose a Wilkinson PD with improved harmonic rejection performance, in^13^. Each of the presented PDs in^12^ and^13^, however, has a large occupied area. In^14^ and^15^, filtering PDs (FPDs) employing stepped impedance resonators have been introduced. Each section of the designed PD in^15^ is composed of three coupled sections. In spite of attaining a decent roll-off in this work, its harmonic suppression performance is not satisfactory. To expand the PD stopband in^16^, five transmission zeros through employing hook-shaped strips have been generated, but using this method has affected both the insertion loss and the roll-off, negatively. In another effort in^17^, coupled quarter-wavelength transmission lines have been utilized to introduce a PD capable of suppressing up to the seventh spurious harmonics with an acceptable bandwidth. However, the suppression level of the harmonics is low. In^18^, another PD which is composed of hairpin-shaped resonators with 6 VIAs has been presented. Two quadratic wavelength resonators with symmetric coupling configuration to enhance the cut-off band have been utilized in^19^. Unfortunately, the proposed structures in^18^ and^19^ suffer from high insertion losses and low number of omitted harmonics. In^20–23^, lowpass filters have been applied to the conventional power divider, which have resulted in a large occupied area. In^20^ and^22^, by utilizing two inhomogeneous coupled lines and the coupling effect of two transmission zeros, respectively, only second and third harmonics have been suppressed. Unfortunately, the suppression level of the PD in^22^ is not satisfactory. In order to achieve unequal power division ratio at the output ports, unequal WPD (UWPD) can be utilized^24–27^. In^24^ and^25^, two UWPDs with 1:2 power division ratios have been introduced, but each of these structures suffers from a very large occupied area. The presented UWPD in^25^ employing electromagnetic bandgap as a high-impedance TL has failed in obtaining a satisfactory return loss value. The UWPDs with 1:4 power-dividing ratio adopting simple microstrip TLs with unequal impedances have been introduced in^26^ and^27^. Unfortunately, employing these methods not only has enlarged the overall circuit size in each case, but also has not been able to suppress unwanted harmonics. In^28^, a new method employing a resistor-inductor-capacitor isolation network to design a filtering power divider (FPD) has been reported. Although using lumped elements to design the proposed divider has been discussed analytically, no lumped elements, such as inductors or capacitors have been applied practically as only microstrip lines have been used in the fabricated circuit. Regardless of the fabrication of the presented FPD in^28^, the parallel inductor and capacitor have been used between output ports to obtain filtering response, but a satisfactory suppression performance has not been obtained thanks to suppressing only the second harmonic. In fact, by applying this lumped network, the isolation between the output ports (S32) has been improved. Moreover, employing this technique has resulted in designing a WPD, which its occupied area is even larger than the conventional WPD introduced by E.J Wilkinson in 1960. In another effort reported in^29^, two capacitors in parallel with the transmission lines of the WPD have been employed to bring about a new zero-reflection frequency and a wider bandwidth in comparison to the conventional WPD. Unfortunately, the introduced structure has failed in noticeable size reduction and also harmonic suppression. In this case, 23% size reduction has been obtained and only the second harmonic has been suppressed. In^30^, by utilizing hybrid design technique composed of lumped elements and microstrip TLs, a WPD capable of harmonic suppression has been presented. However, its operating frequency cannot be changed optionally without redesigning and reconstructing the circuit. According to the presented WPDs operating at 0.8 GHz and 2.4 GHz and their different dimensions, to change the operating frequency, the proposed design has to be redesigned and reconstructed. On the basis of the reported results, the presented WPD in^30^ has been designed to operate as an equal power divider without capability of dividing power at its output ports unequally. In addition to the carried-out investigations on the basis of employing inductors and capacitors in the WPD structure, several reconfigurable^31^ and tunable WPDs utilizing varactors combined with microstrip TLs have been presented so far^32–35^. In spite of their noticeable structures and performances, these WPDs have not been able to suppress spurious frequencies. Moreover, no capability of unequal power division in the introduced WPDs in^32–35^ have been reported. Another drawback of the presented designs in^31–35^ is that each of them has a very large occupied area. In term of unequal power division, two designs utilizing lumped elements^36^ and varactors^37^ have been presented. In^37^, to improve the isolation between output ports, a resistor and a compensative capacitor have been utilized. Then to achieve unequal power division a lumped inductor has been applied to each output port. Employing this technique has brought about 75% size reduction and 2:1 power division. This structure, however, has failed in harmonic suppression and controlling both operating frequency and power division ratio. In^37^, two varactor-loaded L-shaped open-ended stubs have been adopted to modify the performance of the conventional WPD and design a power divider capable of tuning the power division ratio from 1:1 to 1:2.3. However, employing this technique has resulted in unsatisfactory performance in isolation between output ports, matching at all ports and occupying a large area. In this paper, by employing a pair of modified π-shaped resonators consisting of both microstrip TLs and lumped elements a miniaturized WPD capable of suppressing a wide range of spurious harmonics is presented. The operating frequency and also the power division ratio can be selected optionally via changing the values of the lumped elements while the dimensions of the utilized microstrip TLs are kept unchanged. According to the performed analysis, a compact WPD at several operating frequencies of 0.5, 1, 1.5 and 2 GHz is designed and simulated. This divider not only is able to omit spurious frequencies, but also its power division ratio at each operating frequency can be chosen to be equal or unequal. To validate the efficiency of the introduced method, another WPD with equal and unequal power divisions and harmonic suppression at 700 MHz and 1.2 GHz, respectively, has been designed and implemented. According to the measurement results of each case, the 2nd to the 25th harmonics at 700 MHz and the 2nd to 15th harmonics at the second operating frequency have been suppressed. Moreover, almost 96% and 93% size reduction at 700 MHz and 1.2 GHz, respectively, have been obtained. To shift the operating frequency of the fabricated equal WPD at 700 MHz to 1.2 GHz and also achieve unequal power division at the second operating frequency, just the utilized inductors and capacitors have been changed without manipulating the microstrip TLs dimensions and structures.

## The procedure of designing the equal and unequal WPDs

**Figure 1:**
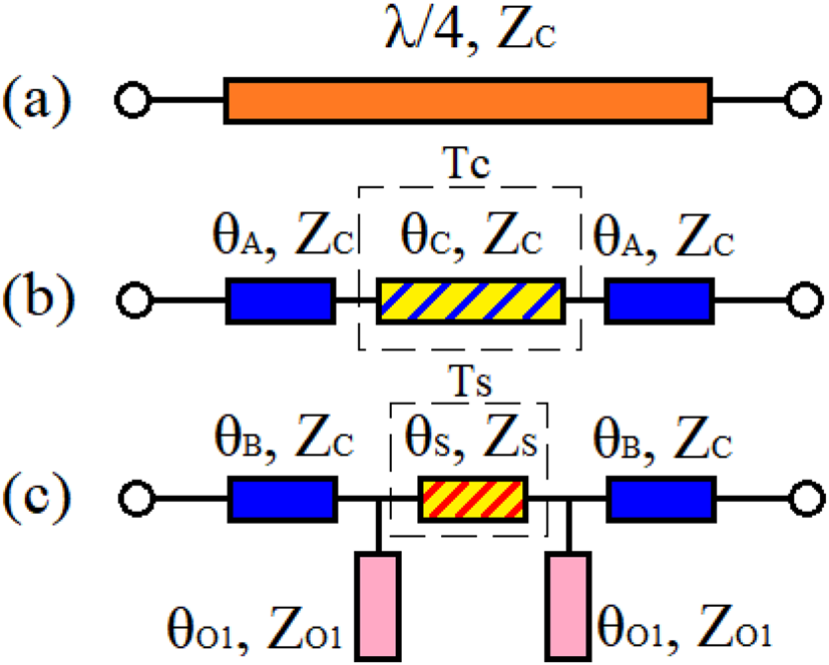
(**a**) Conventional quarter-wavelength TL, (**b**) the separated crosshatched TL determined by T_C_ (*l*_*A*_ + *l*_*C*_ + *l*_*A*_ = *λ/4*) and (**c**) the equivalent π-shaped resonator with open stubs.

In this section, the procedure of replacing a conventional λ/4 transmission line, which is illustrated in Fig. 1a, with a modified π-shaped resonator composed of both microstrip TLs and lumped elements are explained. Then, how this replacement can lead to reducing the circuit size, controlling the magnitude of S21 and choosing the operating frequency, is explained step by step.

### Employing microstrip π-shaped resonator instead of part of λ/4 transmission line

The conventional λ/4 TL is illustrated in Fig. 1a. In the first step, a part of the λ/4 line, which is determined by (*θ*_*C*_*, Z*_*C*_) in Fig. 1b, is replaced by the illustrated microstrip π-shaped resonator in Fig. 1c. The ABCD-matrix of the TLs determined by *T*_*C*_ and *T*_*s*_ in Fig. 1b and c are expressed as

1a $$T_{C} = \left[ {\begin{array}{*{20}c} {\cos \left( {\theta_{c} } \right)} & {jZ_{c} \sin \left( {\theta_{c} } \right)} \\ {\frac{{j\sin \left( {\theta_{c} } \right)}}{{Z_{c} }}} & {\cos \left( {\theta_{c} } \right)} \\ \end{array} } \right]$$

1b $$T_{s} = \left[ {\begin{array}{*{20}c} {\cos \left( {\theta_{s} } \right)} & {jZ_{s} \sin \left( {\theta_{s} } \right)} \\ {\frac{{j\sin \left( {\theta_{s} } \right)}}{{Z_{s} }}} & {\cos \left( {\theta_{s} } \right)} \\ \end{array} } \right]$$

The equivalent matrix of the open stubs *T*_*OS*_ in Fig. 1c is given as

2 $$T_{OS} = \left[ {\begin{array}{*{20}c} 1 & 0 \\ {jB_{O1} } & 1 \\ \end{array} } \right]$$

where the input admittance of the open stubs is

3 $$jB_{O1} = j\frac{{\tan \left( {\theta_{O1} } \right)}}{{Z_{O1} }}$$

The ABCD-matrix of the illustrated π-shaped resonator with open stubs in Fig. 1c can be extracted as follows:

4 $$T_{\pi } = T_{OS} \times T_{s} \times T_{OS}$$

Therefore,

5 $$T_{\pi } = \left[ {\begin{array}{*{20}c} {\cos \left( {\theta_{S} } \right) - B_{O1} Z_{S} \sin \left( {\theta_{S} } \right)} & {jZ_{S} \sin \left( {\theta_{S} } \right)} \\ {j\left( {2B_{O1} \cos \left( {\theta_{S} } \right) - B_{O1}^{2} Z_{S} \sin \left( {\theta_{S} } \right) + \frac{{\sin \left( {\theta_{S} } \right)}}{{Z_{S} }}} \right)} & { - B_{O1} Z_{S} \sin \left( {\theta_{S} } \right) + \cos \left( {\theta_{S} } \right)} \\ \end{array} } \right]$$

According to Fig. 1b and c, the matrix determined by *T*_*C*_ has to be equal to (5); thus, the relations between the parameters of TLs determined by *Tc* and *Ts* in Fig. 1b and c can be obtained by (6–9), as follows:

6 $$\cos \left( {\theta_{C} } \right) = \cos \left( {\theta_{S} } \right) - B_{O1} Z_{S} \sin \left( {\theta_{S} } \right)$$

7 $$Z_{C} \sin \left( {\theta_{C} } \right) = Z_{S} \sin \left( {\theta_{S} } \right)$$

8 $$\frac{{\sin \left( {\theta_{C} } \right)}}{{Z_{C} }} = 2B_{O1} \cos \left( {\theta_{S} } \right) - B_{O1}^{2} Z_{S} \sin \left( {\theta_{S} } \right) + \frac{{\sin \left( {\theta_{S} } \right)}}{{Z_{S} }}$$

9 $$\cos \left( {\theta_{C} } \right) = - B_{O1} Z_{S} \sin \left( {\theta_{S} } \right) + \cos \left( {\theta_{S} } \right)$$

*θ*_*S*_ can be defined as

10 $$\theta_{S} = \alpha \theta_{C}$$

where (α) can be chosen as a value between 0 and 1 (0 < α < 1). According to Fig. 1b, the value of *θ*_*C*_ is *θ*_*C*_ < π/2.

Consequently, the value of *Z*_*S*_ can be calculated from (7) as

11 $$Z_{S} = \frac{{Z_{C} \sin \left( {\theta_{C} } \right)}}{{\sin \left( {\theta_{S} } \right)}}$$

From (3), (6) and (7),

12 $$\theta_{O1} = \tan^{ - 1} \left( {\frac{{Z_{O1} }}{{Z_{C} }} \times \frac{{\cos \left( {\theta_{S} } \right) - \cos \left( {\theta_{C} } \right)}}{{\sin \left( {\theta_{C} } \right)}}} \right)$$

When a part of the conventional λ/4 TL is selected to be replaced by a π-shaped resonator with open stubs, the values of θ_C_ and Z_C_ in (12) are determined. By selecting the value of α, the value of θ_s_ can be obtained from (10). Thus, the equation determined by (12) can describe the relation between *θ*_*O1*_ and *Z*_*O1*_.

**Figure 2:**
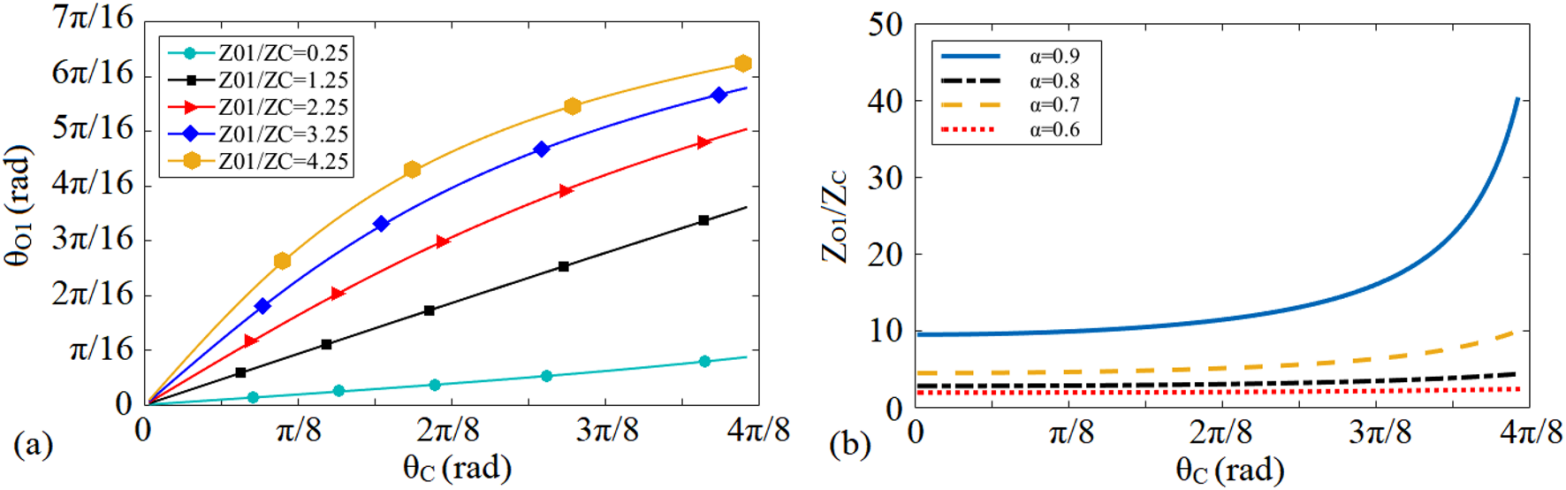
(**a**) Variations of *θ*_*01*_ and (**b**) *Z*_*O1*_/*Z*_*C*_ versus *θ*_*C*_.

As shown in Fig. 2a, while a low value of the illustrated *θ*_*C*_ in Fig. 1b is chosen to be replaced by the shown π-shaped resonator in Fig. 1c, open stubs with lower electrical lengths and characteristic impedances are needed; consequently, a π-shaped resonator with open stubs with a lower occupied area is obtained. Moreover, according to Fig. 2a, utilizing open stubs with lower value of *Z*_*O1*_/* Z*_*C*_ can result in more size reduction. According to Fig. 2b, for lower values of (*α*), microstrip open stubs with lower characteristic impedances are needed.

### Employing high-low impedance stubs instead of the open stubs of microstrip π-shaped resonator

**Figure 3:**
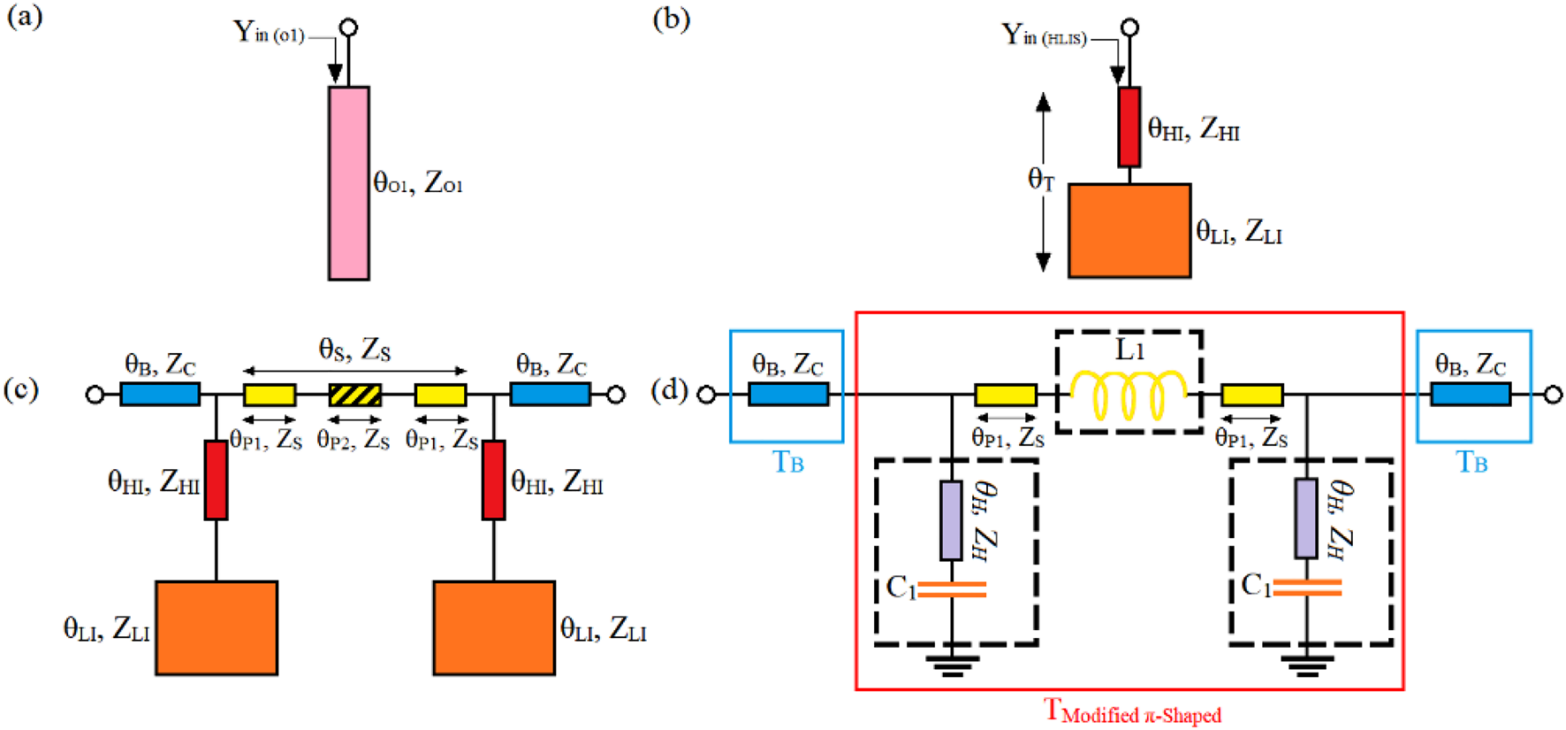
(**a**) The open stub, (**b**) its equivalent HLIS and (**c**) microstrip π-shaped resonator with high-low impedance stubs (**d**) the modified π-shaped resonator with combined microstrip TL and lumped element.

**Figure 4:**
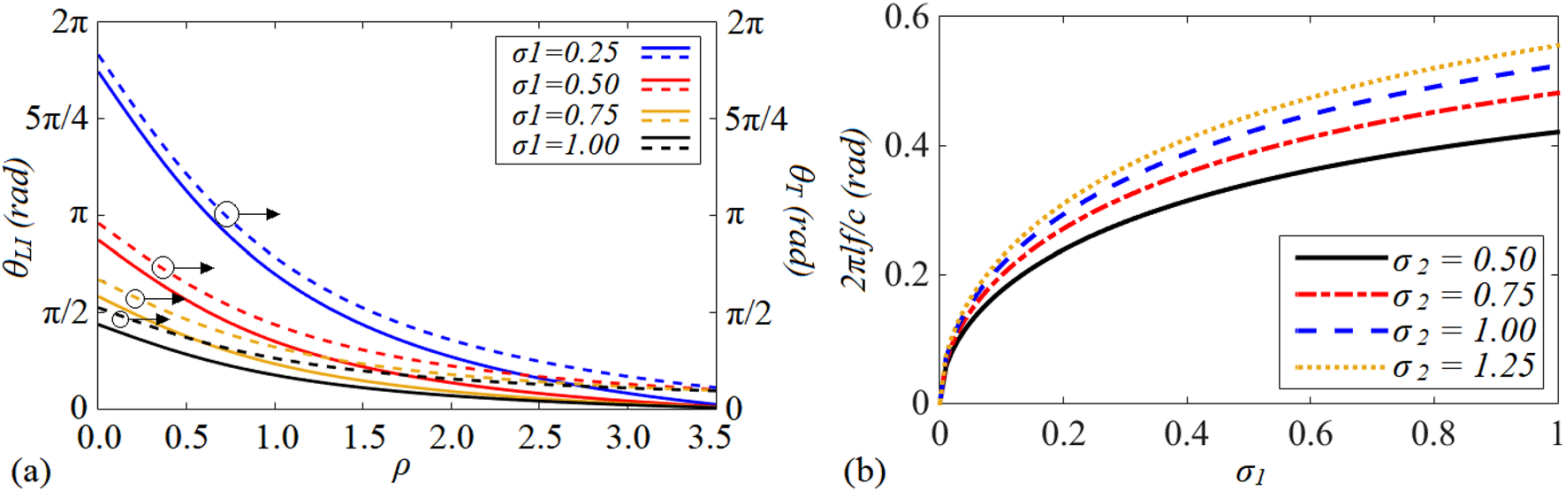
(**a**) The total electrical length *θ*_*T*_ versus *(ρ)* and the electrical length *θ*_*LI*_ versus (*ρ)* for different values of (*σ*_*1*_) and (**b**) the variation of the normalized -3 dB operating frequency of the shown cell in Fig. 3c (*θ*_*s*_ = *2θ*_*HI*_ = 2*θ*_*LI*_ = 2*θ*) versus *σ*_*1*_ = *(Z*_*LI*_*/Z*_*HI*_*)* for different values of *σ*_*2*_ = *(Z*_*HI*_*/Z*_*s*_*).*

In the second step, to decrease the occupied area even more, the depicted high-low impedance stubs (HLISs) in Fig. 3b can be utilized instead of the open stubs of the shown π-shaped resonator in Fig. 1c. The following lines clarifies how utilizing HLIS instead of the shown open stub in Fig. 3a can lead to size reduction. The electrical lengths and characteristic impedances of the high impedance and low impedance TLs of the HLIS are determined by (*θ*_*HI*_*, Z*_*HI*_) and (*θ*_*LI*_*, Z*_*LI*_), respectively, as illustrated in Fig. 3b. Equating *Y*_*in (O1)*_ with *Y*_*in (HLIS)*_ yields:

13 $$\sigma_{1} \cot \left( {\uptheta _{LI } } \right) = \frac{{\uprho + \tan (\theta_{{{\text{HI}}}} )\tan (\theta_{{{\text{O1}}}} )}}{{\tan (\theta_{{{\text{O1}}}} ) - \rho \tan (\theta_{{{\text{HI}}}} )}}$$

where* σ*_*1*_ = *Z*_*(LI)*_/*Z*_*(HI)*_ and *ρ* = *Z*_*(O1)*_*/Z*_*(HI)*_. The variation of the *θ*_*LI*_ versus the ratio of (*ρ)* for different values of *(σ*_*1*_*)* has been illustrated in Fig. 4a. According to Fig. 4a, when the values of the ratios determined by (*ρ)* and (*σ*_*1*_) enhance, the electrical length of the low impedance TL i.e., *θ*_*LI*_ decreases and consequently the total electrical length of HLIS reduces. This means that by adopting higher values of (*ρ)* and *σ*_*1*_, a high-low impedance stub with a lower total length in comparison to the shown open stub in Fig. 3a can be obtained. Moreover, as illustrated in Fig. 4a, no significant difference between the values of *θ*_*LI*_ and *θ*_*T*_ (*θ*_*T*_ = *θ*_*LI*_ + *θ*_*HI*_) which are plotted versus the ratio of (*ρ* = *Z*_*(O1)*_*/Z*_*(HI*_*)* can be seen. This means that, while transferring the shown open-stub in Fig. 3a to the depicted high-low impedance resonator in Fig. 3b, the impact of the electrical length of the high-impedance line (*θ*_*HI*_) is trivia. Therefore, to decrease the occupied area, high-impedance line with the minimum value of *θ*_*HI*_ and consequently the lowest possible physical length can be employed. In this case, the lowest possible physical length, which depends on the accuracy of the fabrication process, is the minimum length (or width) of the microstrip line which can be implemented by the manufacturer. In this manuscript, the lowest possible physical length is *L*_*HI (minimum)*_ = 0.1 mm. Employing HLIS instead of the open stubs of the shown π-shaped resonator in Fig. 1c leads to obtaining a π-shaped resonator with high-low impedance stubs, as illustrated in Fig. 3c. Note that owing to the lowpass filtering characteristics of the π-shaped resonator with HLISs^38,39^, this resonator can add the capability of suppressing spurious frequencies to the final design of the WPD.

### Calculating a relation between the operating frequency of the π-shaped resonator with HLISs and its transmission lines’ characteristic impedances

To clarify how the characteristic impedances of the presented π-shaped resonator with HLISs in Fig. 3c i.e., *Z*_*HI*_, *Z*_*LI*_ and *Z*_*s*_ can be exploited to control the − 3 dB operating frequency, their corresponding electrical lengths can be assumed equal to *θ*_*s*_ = *2θ*_*HI*_ = 2*θ*_*LI*_ = 2*θ*. Note that, *θ* = *βl* where *β* and *l* are the propagation constant and physical length of each microstrip TL, respectively. By following the same process reported in^40^, the − 3 dB cut-off frequency of the shown resonator in Fig. 3c can be extracted as follows:

14 $$f = \frac{c}{2\pi l}\tan^{ - 1} \left( {\sqrt {\frac{{\sigma_{1} \sigma_{2} }}{{\sigma_{1} + \sigma_{2} + 1}}} } \right)$$

where *σ*_*1*_ = *(Z*_*LI*_/*Z*_*HI*_*)* and *σ*_*2*_ = *(Z*_*HI*_/*Z*_*s*_*)*. According to (14), the − 3 dB operating frequency as a function of (*σ*_*1*_*)* and (*σ*_*2*_*)* can be changed optionally, as depicted in Fig. 4b. As can be seen, in order to achieve a desired operating frequency, the ratios determined by *σ*_*1*_ = *(Z*_*LI*_/*Z*_*HI*_*)* and *σ*_*2*_ = *(Z*_*HI*_/*Z*_*s*_*)* can be tuned.

### Employing lumped elements in the structure of the π-shaped resonator with HLIS and investigating its impacts on the resonator performance and its size

In the third step, to decrease the overall occupied area and also being able to control the − 3 dB cut-off frequency without changing the physical lengths and characteristic impedances of the microstrip lines, the depicted high-low impedance stubs and their connecting TL determined by *(θ*_*LI*_*, Z*_*LI*_*)* and *(θ*_*P2*_*, Z*_*S*_*)* in Fig. 3c are replaced by their equivalent lumped elements. Consequently, the depicted modified π-shaped resonator with combined microstrip TL and lumped element in Fig. 3d is obtained. The illustrated *C*_*1*_ in Fig. 3d is the equivalent capacitor of the low-impedance TL determined by *(θ*_*LI*_*, Z*_*LI*_*)* in Fig. 3c, which can be calculated by^39^:

15 $$C_{1} = \frac{{l_{LI} }}{{Z_{LI} \times \nu_{p} }}$$

This means that instead of the low-impedance of the introduced HLIS, a lumped capacitance which its value can be obtained by (15) can be employed.

Moreover, the depicted *L*_*1*_ in Fig. 3d is equivalent inductance of the microstrip TL determined by *(θ*_*P2*_*, Z*_*S*_*)* in Fig. 3c, which can be extracted by^39^:

16 $$L_{1} = \frac{{l_{P2} \times Z_{S} }}{{\nu_{p} }}$$

A lumped inductor, which its value can be calculated by (16), can be used instead of the connecting TL of the introduced π-shaped resonator with HLISs. Thus, instead of decreasing the operating frequency by increasing the physical length of the connecting TL determined by (*l*_*p2*_) in Fig. 3c, which leads to enhancing the circuit size, the value of this lumped inductor can be increased.

Note that, as the large dimensions of the microstrip low-impedance line and also the long length of the microstrip connecting line determined by *(θ*_*LI*_*, Z*_*LI*_*)* and *(θ*_*P2*_*, Z*_*S*_*)*, respectively, require a very large area, employing their corresponding lumped elements defined by *C*_*1*_ and *L*_*1*_ instead of them can decrease the occupied area considerably, as explained.

To clarify how the overall occupied area, the operating frequency and also the magnitude of the insertion loss (S21) of the modified resonator illustrated in Fig. 3d can be controlled by the variations of just lumped elements determined by *L1* and *C1*, calculating the relation of transmission coefficient (S21) can be helpful. This relation can be extracted from Fig. 3d as follows^39^:

17 $$S_{21} = \frac{2}{{A_{Total} + {{B_{Total} } \mathord{\left/ {\vphantom {{B_{Total} } {Z_{0} }}} \right. \kern-0pt} {Z_{0} }} + C_{Total} Z_{0} + D_{Total} }}$$

18 $$T_{Total} = T_{B} \times T_{Modified \pi - Shaped} \times T_{B}$$

where

19 $$T_{B} = \left[ {\begin{array}{*{20}c} {\cos \left( {\theta_{B} } \right)} & {jZ_{i} \sin \left( {\theta_{B} } \right)} \\ {\frac{{j\sin \left( {\theta_{B} } \right)}}{{Z_{B} }}} & {\cos \left( {\theta_{B} } \right)} \\ \end{array} } \right]$$

And also, the matrix determined by $T_{Modified \pi - Shaped}$ and its parameters can be obtained as

20a $$T_{Modified \pi - Shaped} = \left[ {\begin{array}{*{20}c} {A_{Modified \pi - Shaped} } & {B_{Modified \pi - Shaped} } \\ {C_{Modified \pi - Shaped} } & {D_{Modified \pi - Shaped} } \\ \end{array} } \right]$$

20b $$A_{Modified \pi - Shaped} = D_{Modified \pi - Shaped} = \frac{{\upsilon_{p} + \left( {C_{1} Z_{H} l_{H} + \upsilon_{p} C_{1} L_{1} + 2l_{P1} Z_{P1} C_{1} } \right)s^{2} }}{{\upsilon_{p} + C_{1} Z_{H} l_{H} s^{2} }}$$

20c $$B_{Modified \pi - Shaped} = L_{1} s + \frac{{2l_{P1} Z_{P1} s}}{{\upsilon_{p} }}$$

20d $$C_{Modified \pi - Shaped} = \frac{{\upsilon_{p} C_{1} s\left[ {2\upsilon_{p} + \left( {2C_{1} Z_{H} l_{H} + \upsilon_{p} C_{1} L_{1} + l_{P1} Z_{P1} C_{1} } \right)s^{2} } \right]}}{{\left( {\upsilon_{p} + C_{1} Z_{H} l_{H} s^{2} } \right)^{2} }}$$

where *v*_*P*_ represents the phase velocity.

**Figure. 5:**
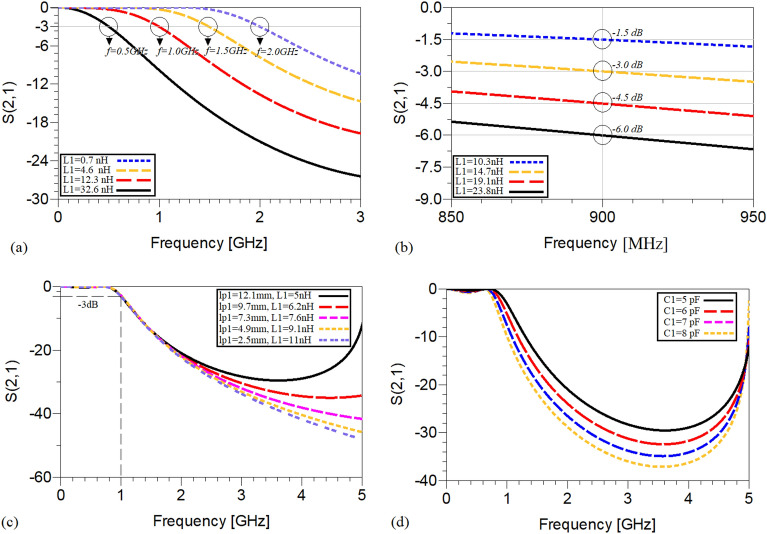
The variation of S21 (**a**) versus L1 to control the operating frequency at a fixed S21 level, (**b**) versus *L1* to control the level of S21 at a fixed operating frequency of 900 MHz, (**c**) versus L1 and the physical length determined by *l*_*p1*_ to show size reduction and (**d**) versus *C1*.

According to (17)–(20), the S21 as a function of several variables is plotted, as depicted in Fig. 5a–d. According to Fig. 5a and b, both the operating frequency and the level of the S21 can be controlled only via changing the value of the inductance determined by *L1*, while the other variables such as *W*_*S*_, *l*_*p1*_, *l*_*H*_, *Z*_*H*_, *l*_*B*_ and *C1* are kept constant. The values of these variables are equal to 1 mm, 12.1 mm, 0.1 mm, 115 Ω, 2.3 mm and 2pF, respectively. Note that, the value of *Z*_*H*_ is calculated based on a microstrip high-impedance line with length and width of 0.1 mm and 0.2 mm, respectively, and a 0.504 mm-thickness substrate with the permittivity of 3.38 and the loss tangent of 0.00027^39^.

Obviously, according to Fig. 5a and b, while the value of *L1* is increased and simultaneously the physical length of the microstrip TL line determined by *l*_*p1*_ is decreased, both the operating frequency and the level of S21 remain unchanged; thus, by following this tuning process which includes two steps i.e., (a) declining the physical length determined by *l*_*p1*_ gradually and (b) enhancing the value of *L1* (to compensate for the decrease of *l*_*p1*_), results in a remarkable size reduction without affecting the operating frequency and the level of S21. The obtained size reduction in this case is almost 80%. Moreover, as illustrated in Fig. 5d, enhancing the value of *C1* with steps of (1.0) leads to improving the stop band rejection level considerably and decreasing the − 3 dB operating frequency, to some extent.

**Figure 6:**
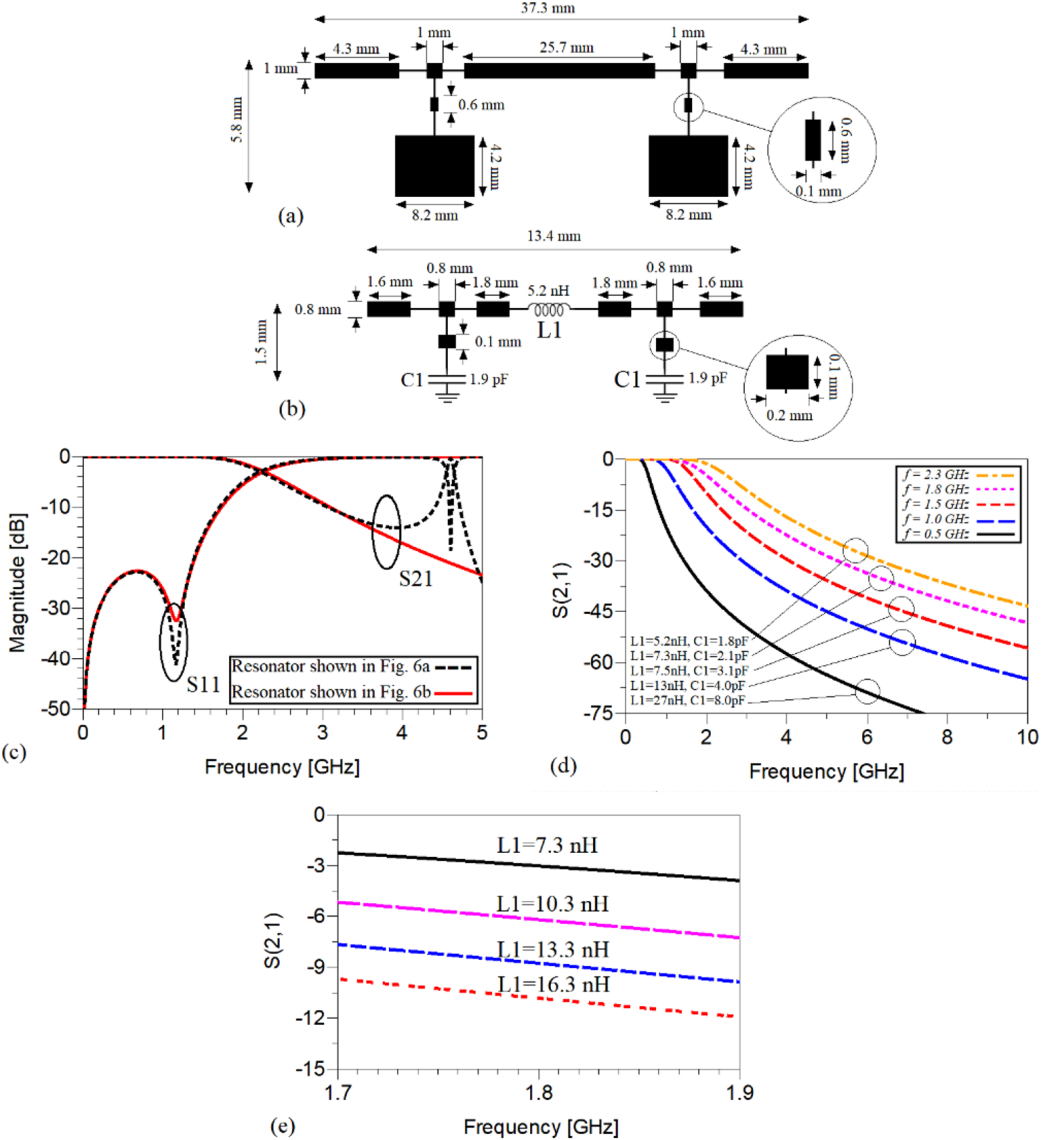
The microstrip realization of the shown cell (**a**) in (**b**) (**c**) their frequency response (**d**) performance of the modified π-shaped resonator with combined microstrip TL and lumped element at different operating frequencies and (**e**) variation of the S21 versus changing the value of just L1.

A microstrip realization of the illustrated π-shaped resonator with high-low impedance stubs in Fig. 3c and its equivalent modified π-shaped resonator consisting of microstrip TLs and lumped elements which is shown in Fig. 3d with an operation frequency of 2.2 GHz are depicted in Fig. 6a and b, respectively. The agreement between the frequency responses of π-shaped resonator with high-low impedance stubs and its modified version composed of microstrip TLs and lumped elements, which is shown in Fig. 6c, confirms that the resonator with lumped elements is an acceptable substitution of the resonance cell shown in Fig. 6a. By comparing the dimensions of the shown resonators in Fig. 6a and b, it can be observed that while employing the lumped elements, the occupied area of the resonator decreases considerably, as expected. As shown in Fig. 6d, the modified π-shaped resonator in Fig. 6b can be tuned to operate at different frequencies via changing the values of its lumped elements i.e., *L1* and *C1*. It means that, to redesign this cell to operate at another operating frequency, the dimensions of the employed microstrip TLs can be kept unchanged and just the values of lumped inductance and capacitances need to be varied. Moreover, according to Fig. 6e, the magnitude of S21 can be controlled via changing the value of the inductance *L1*. As can be observed, by increasing the value of *L1* from 7.3 to 16.3 nH with steps of 3, the magnitude of S21 changes from − 3.023 to − 10.28 dB at the operating frequency of 1.8 GHz. Note that, the frequency responses and explanations obtained from Fig. 6 confirm the depicted results in Fig. 5.

### Applying the designed π-shaped resonator with lumped elements to design miniaturized equal/unequal WPDs with harmonic suppression

**Figure 7:**
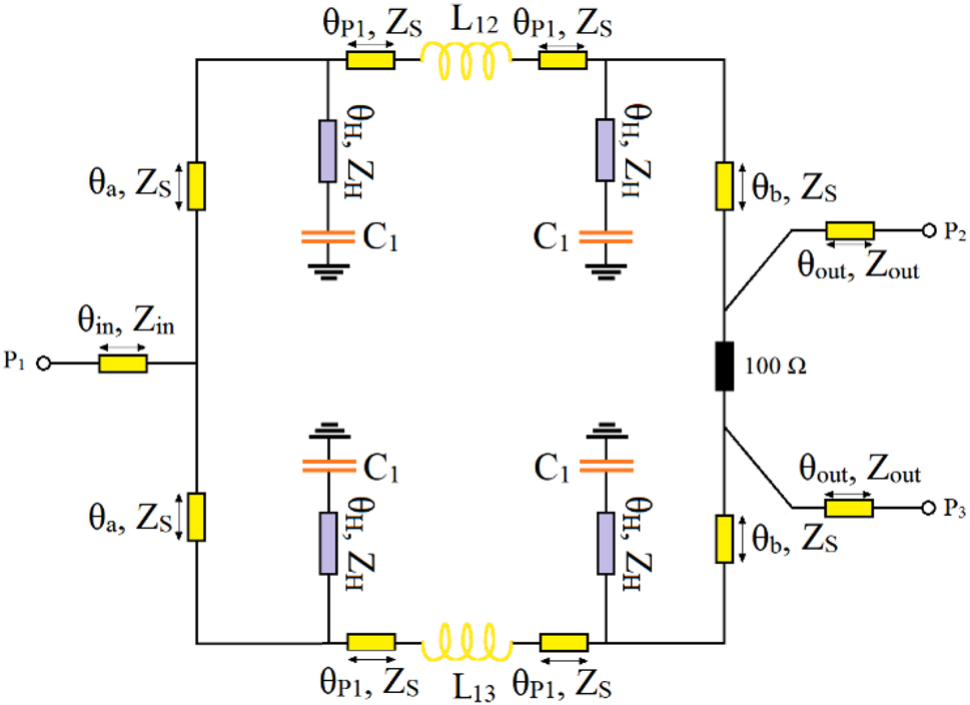
The configuration of the proposed WPD.

In the final step, a WPD utilizing the designed π-shaped resonator with lumped components is introduced. By applying the modified π-shaped resonator depicted in Fig. 3d to the conventional WPD introduced in^39^, a miniaturized power divider which not only is able to operate at different frequencies with optional equal or unequal power division, but also is capable of suppressing spurious frequencies over a very wide range is designed and simulated. The schematic of the proposed WPD is illustrated in Fig. 7.

As can be seen, the proposed WPD is completely symmetrical around X-axis, apart from the values of the connecting inductances determined by *L*_*12*_ and *L*_*13*_. These inductors can be used to determine the power division ratio at the output ports; when the values of *L*_*12*_ and *L*_*13*_ are equal, an equal power division at output ports is attained and if *L*_*12*_ ≠ *L*_*13*_ then *P*_*2*_* ≠ P*_*3*_. The other lumped elements and TLs characteristics employed in the two branches of the WPD, however, are kept equal, as shown in Fig. 7.

To show the ability of the proposed WPD in operating at different frequencies with optional power division ratio at each operating frequency, several samples operating at 0.5, 1.0, 1.5 and 2 GHz are introduced. The dimensions of the utilized microstrip TLs of the proposed WPD with equal power division ratio at the output ports at all chosen operating frequencies are: W_S_ = 1.5 mm, L_a_ = 1.5 mm, L_P1_ = 3.6 mm, L_b_ = 1.4 mm, L_H_ = 0.1 mm and W_H_ = 0.2 mm; the values of capacitors and inductor at 0.5 GHz are L_13_ = L_12_ = 20 nH and C1 = 4.2 pF, at 1.0 GHz are L_13_ = L_12_ = 10 nH and C1 = 1.6 pF, at 1.5 GHz are L_13_ = L_12_ = 5.6 nH and C1 = 1 pF and at 2 GHz are L_13_ = L_12_ = 4.7 nH and C1 = 0.7 pF.

**Figure 8:**
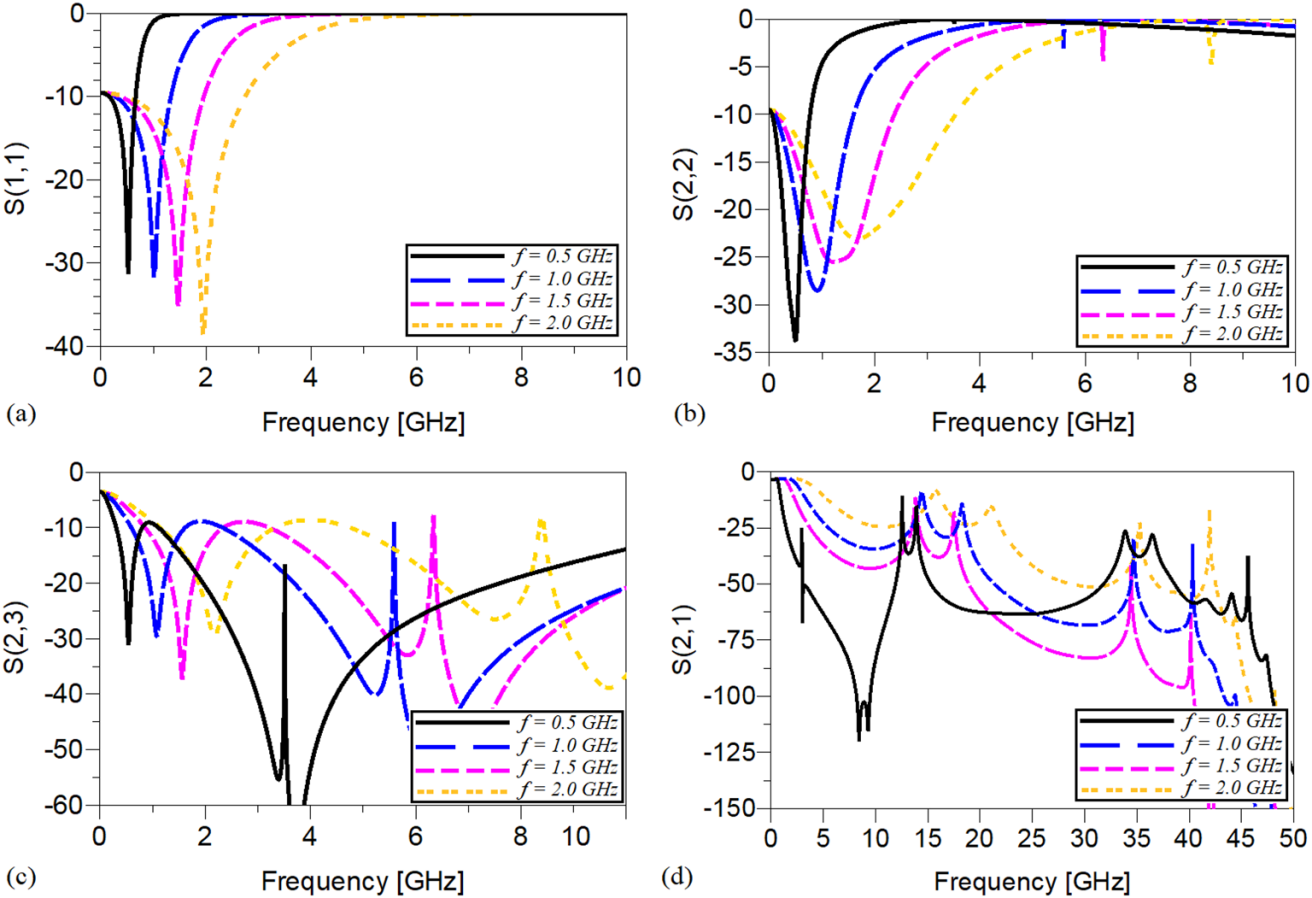
The frequency response of the proposed Wilkinson power divider with equal power division ratios at the output ports at 0.5, 1.0, 1.5 and 2 GHz (**a**) S11, (**b**) S22, (**c**) S23 and (**d**) S21.

Obviously, to change the operating frequency, the physical lengths and widths of the utilized microstrip TLs have not been manipulated and kept constant. It means that, by tuning the values of just the employed lumped elements determined by *L*_*13*_, *L*_*12*_ and *C*_*1*_ the operating frequency can be controlled. The scattering parameters of the shown WPD in Fig. 7 operating at 0.5, 1.0, 1.5 and 2 GHz are depicted in Fig. 8. As illustrated, not only acceptable performances in S11, S22 and S23 have been obtained at the mentioned operating frequencies, but also on the basis of Fig. 8d spurious frequencies over a very wide range have been suppressed. Table 1 summarizes the simulation results of each WPD at the above-mentioned frequencies.

**Table 1:** The simulation results of the designed WPDs at 0.5, 1, 1.5 and 2 GHz with equal power divisions at the output ports (NB: L_min_ = the lowest level of suppression).

Operating frequency	0.5 GHz	1 GHz	1.5 GHz	2 GHz
Size reduction	96.64%	91.85%	82.2%	62.9%
Harmonics suppressed	2nd–100th L_min_ = − 11.620 dB	2nd–50th L_min_ = − 10.830 dB	2nd–34th L_min_ = − 11.014 dB	2nd–25th L_min_ = − 10.02 dB
S_11_	− 26.923 dB	− 41.142 dB	− 27.925 dB	− 35.461 dB
S_21_	− 3.019 dB	− 3.012 dB	− 3.021 dB	− 3.015 dB
S_22_	− 45.038 dB	− 32.511 dB	− 60.694 dB	− 26.993 dB
S_23_	− 23.468 dB	− 28.146 dB	− 24.772 dB	− 22.122 dB

**Figure 9:**
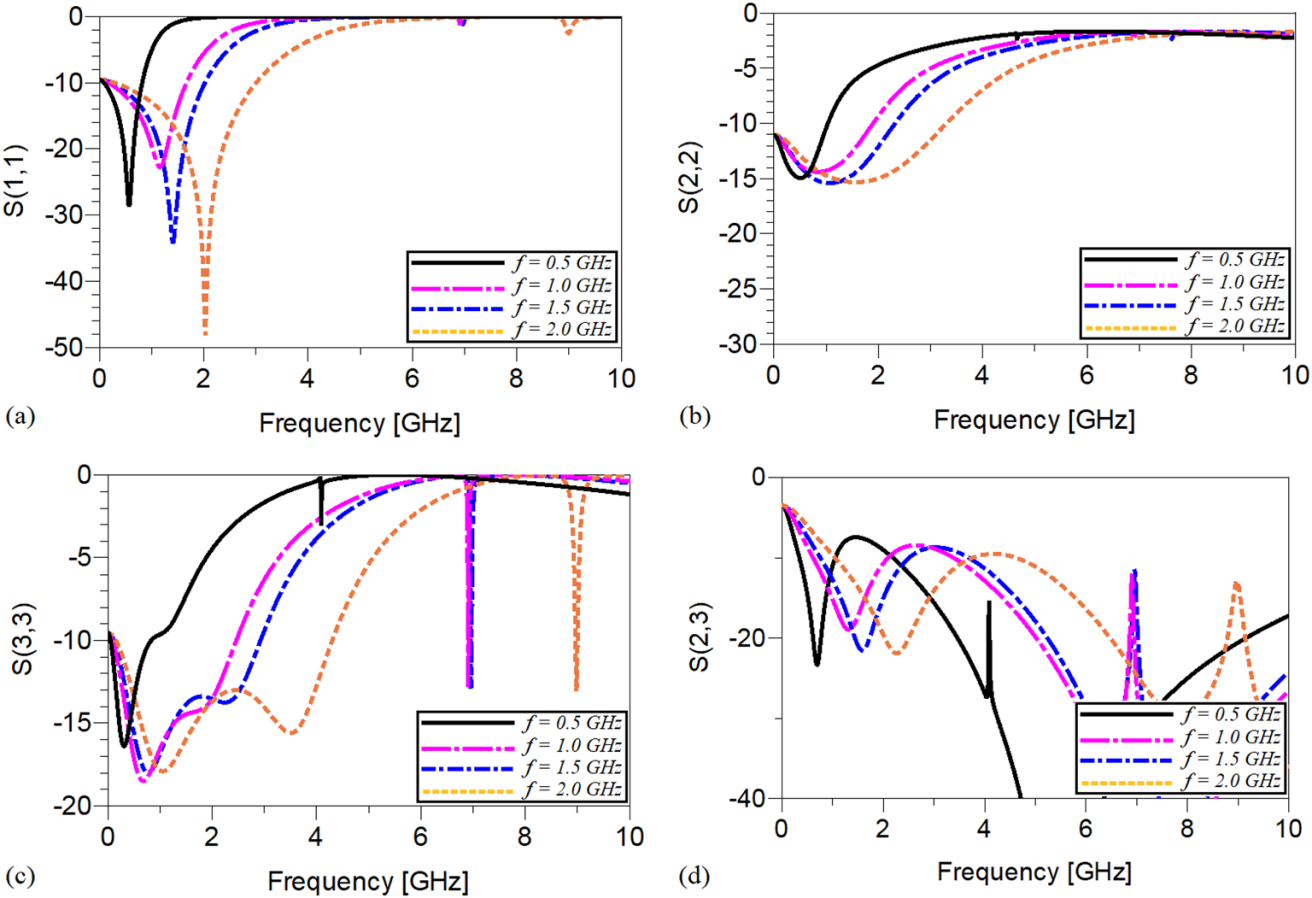
The frequency response of the proposed Wilkinson power divider with unequal power division ratios at its output ports at 0.5, 1, 1.5 and 2 GHz (**a**) S11, (**b**) S22, (**c**) S33 and (**d**) S23.

**Figure 10:**
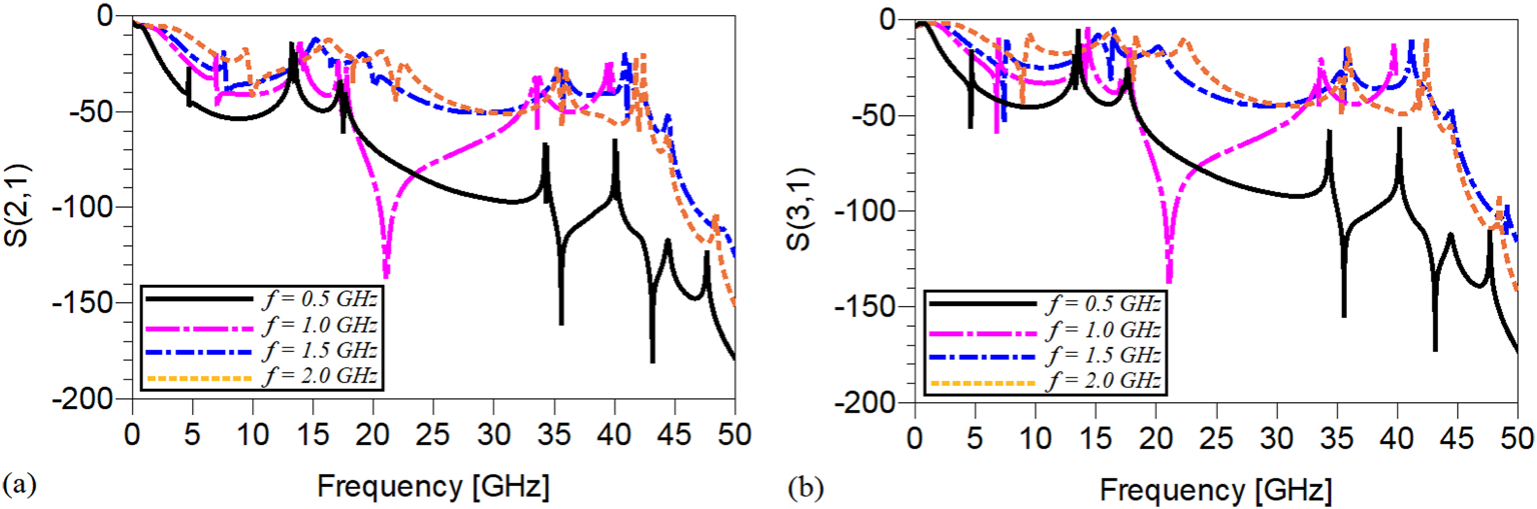
The harmonic suppression performance of the proposed Wilkinson power divider with unequal power division ratios at its output ports at 0.5, 1, 1.5 and 2 GHz (**a**) S21 and (**b**) S31.

**Figure 11:**
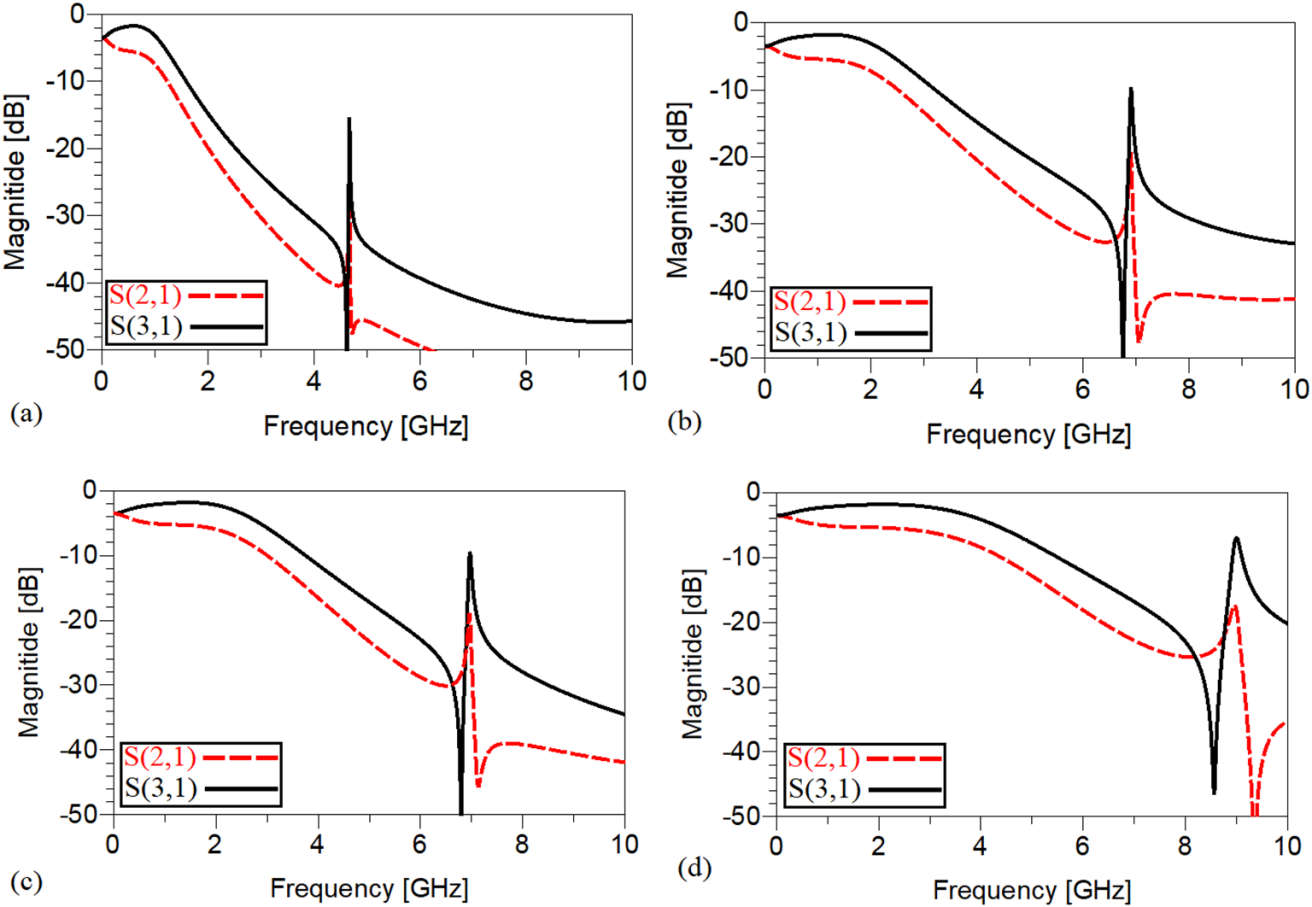
A comparison of the S21 and S31 of the proposed Wilkinson power divider with unequal power division ratios at the output ports at (**a**) 0.5, (**b**) 1, (**c**) 1.5 and (**d**) 2 GHz.

The frequency responses of the presented unequal divider at different operating frequencies are shown in Figs. 9, 10 and 11. As can be observed in Fig. 9, acceptable performances in S11, S22 and S23 have been attained at the chosen operating frequencies. According to Fig. 10, which shows the harmonic suppression performances of S21 and S31, spurious harmonics over a very wide range have been suppressed. To show the unequal power division in each operating frequency more clearly, the S21 and S31 of the simulated WPD are compared in Fig. 11. Table 2 summarizes the simulation results of each unequal WPD at 0.5, 1, 1.5 and 2 GHz operating frequencies. Obviously, on the basis of the illustrated simulations, at the given operating frequencies almost similar power division ratio (2.3:1) can be achieved. The dimensions of the utilized microstrip TLs of the proposed unequal WPD at all chosen operating frequencies are similar to the mentioned values of the equal WPD; the values of the capacitors and inductors, however, are different as at 0.5 GHz are L_13_ = 9 nH, L_12_ = 30 nH and C1 = 2.4 pF, at 1.0 GHz are L_13_ = 4.5 nH, L_12_ = 16nH and C1 = 0.8 pF, at 1.5 GHz are L_13_ = 3.3 nH, L_12_ = 12 nH and C1 = 0.7 pF and at 2 GHz are L_13_ = 1.8 nH, L_12_ = 8.5 nH and C1 = 0.5 pF.

**Table 2:** The simulation results of the designed WPD at 0.5, 1, 1.5 and 2 GHz with unequal power divisions at the output ports (NB: L_min_ = the lowest level of suppression).

Operating frequency	0.5 GHz	1 GHz	1.5 GHz	2 GHz
Size reduction	96.64%	91.85%	82.2%	62.9%
Harmonics suppressed (S_21_)	3rd to more than 100th L_min_ = − 13.530 dB	3rd to more than 50th L_min_ = − 13.491 dB	3rd to more than 34th L_min_ = -18.467 dB	3rd to more than 25th L_min_ = -16.397 dB
Harmonics suppressed (S_31_)	3rd to 100th L_min_ = − 10.023 dB	3rd to 50th L_min_ = − 10.109 dB	3rd to 34th L_min_ = − 13.182 dB	3rd to 25th L_min_ = − 11.084 dB
S_11_	− 29.360 dB	− 22.749 dB	− 33.052 dB	− 40.692 dB
S_21_	− 5.473 dB	− 5.431 dB	− 5.351 dB	− 5.378 dB
S_31_	− 1.805 dB	− 1.842 dB	− 1.817 dB	− 1.808 dB
S_22_	− 15.31 dB	− 14.96 dB	− 15.67 dB	− 16.18 dB
S_33_	− 16.417 dB	− 16.554 dB	− 13.948 dB	− 13.844 dB
S_23_	− 23.2 dB	− 21.36 dB	− 19.023 dB	− 21.636 dB

Note that, to design the equal and unequal WPDs at 0.5, 1.0, 1.5 and 2 GHz, first a power divider with equal power division ratio at the output ports was designed to operate at 0.5 GHz. Then, to design the other equal and unequal WPDs operating at 1, 1.5 and 2 GHz, just the lumped elements of the divider operating at 0.5 GHz were changed, while the dimensions of the whole employed microstrip TLs in this circuit were kept constant. Consequently, the circuit size i.e., length (mm) × width (mm) of each proposed equal/unequal design is remained unchanged. This means that the miniaturization percentage decreases while the operating frequency increases, as shown in Tables 1.

### The calculations of the electrical lengths and characteristic impedances of the employed TLs in the designed WPD

**Figure 12:**
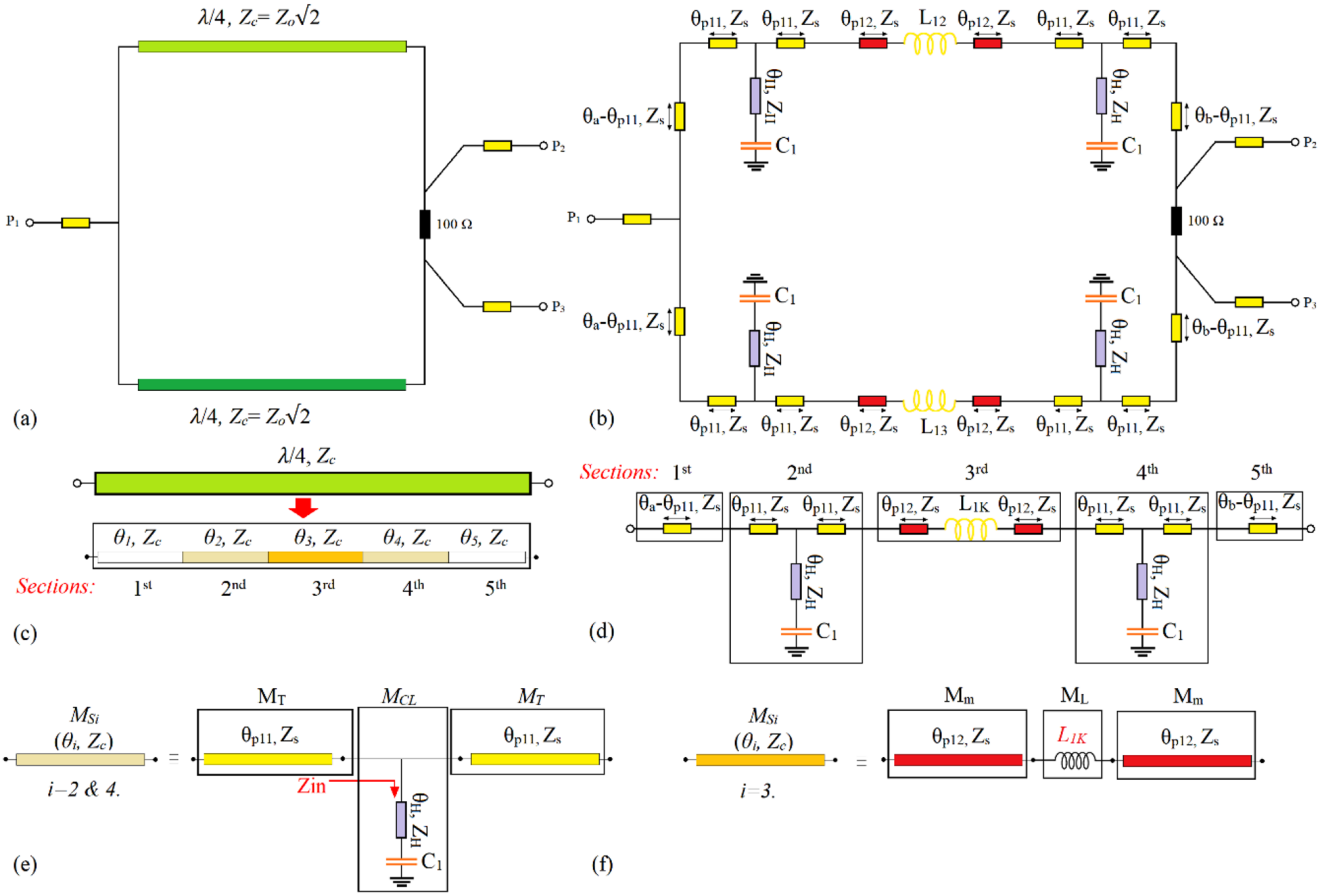
(**a**) The conventional WPD^1^, (**b**) the proposed WPD with divided sections, (**c**) the λ/4 TL employed in the conventional WPD^1^, (**d**) the utilized TL instead of the λ/4 TL, (**e**) the second (or forth) section of the λ/4 TL and its equivalent T-shaped resonator and f) the third section of the λ/4 TL and its modified series structure utilizing a lumped inductor *L*_*1K*_ (*L*_*12*_ or *L*_*13*_).

The configuration of the conventional WPD and the proposed structure with five divided sections, are illustrated in Fig. 12a and b, respectively. The λ/4 TL employed in the conventional WPD with the characteristic impedance of *Z*_*c*_ = *Z*_*o*_√2, where *Z*_*o*_ = 50 Ω, can be divided into five similar sections with equal electrical lengths (*θ*_*1*_ = *θ*_*2*_ = *θ*_*3*_ = *θ*_*4*=_ *θ*_*5*_ = *0.1π* = *θ*_*i*_) and similar characteristic impedances (*Z*_*c*_), as illustrated in Figs. 12c. Each of these sections, which are determined by (*θ*_*i*_*, Z*_*c*_) where i = 1, 2, 3, 4 and 5, are replaced by different structures to design the proposed WPD depicted in Fig. 7, as shown in Fig. 12d. Note that, the subscript (*i*) specifies each of the chosen section of the λ/4 TL in Fig. 12c and its corresponding section in Fig. 12d.

By comparing Fig. 12c and d, it can be assumed that the second and forth sections of the conventional λ/4 TL i.e., (*θ*_*2*_*, Z*_*c*_) and (*θ*_*4*_*, Z*_*c*_) are replaced by modified T-shaped resonators illustrated in Fig. 12e. The corresponding sections of the conventional λ/4 microstrip TL are specified by (*θ*_*i*_*, Z*_*c*_), where *i* = *2* and *4*, and the microstrip TLs of their equivalent modified T-shaped resonators are determined by (*θp*_*11*_*, Z*_*s*_), where to decrease the circuit size, it can be concluded that 2*θp*_*11*_<* θ*_*i*_. Moreover, the employed high-impedance lines and the capacitances in both resonators are determined by (*θ*_*H*_*, Z*_*H*_) and* C*_*1*_. Thus, when *i* = *2* the calculations are related to the second section of the λ/4 TL and its equivalent T-shaped resonator and *i* = *4* determines the calculations relevant to the fourth section of the λ/4 TL and its corresponding T-shaped resonator.

In the first step, the values of *θp*_*11*_ and* Z*_*s*_ as functions of the electrical length and characteristic impedance of the chosen section of the conventional microstrip TL specified by (*θ*_*i*_*, Z*_*c*_*)* and the employed lumped capacitors determined by *C*_*1*_ are calculated. The ABCD matrix of the microstrip TL in Fig. 12e, which is determined by (*θ*_*i*_*, Z*_*c*_), can be expressed as:

21 $$M_{Si} = \left[ {\begin{array}{*{20}c} A & B \\ C & D \\ \end{array} } \right] = \left[ {\begin{array}{*{20}c} {\cos \left( {\theta_{i} } \right)} & {jZ_{c} \sin \left( {\theta_{i} } \right)} \\ {jY_{c} \sin \left( {\theta_{i} } \right)} & {\cos \left( {\theta_{i} } \right)} \\ \end{array} } \right]$$

According to the explanations of Fig. 4a, the physical length of each of the employed high-impedance lines is equal to *L*_*H(minimum)*_ = 0.1 mm, which is the lowest possible physical length to be implemented. This means that the corresponding value of *θ*_*H*_ and consequently, *tag(θ*_*H*_*)* is very close to zero. Accordingly, the depicted input impedance in Fig. 12e i.e., *Z*_*in*_ is equal to *1/jωC*_*1*_. The ABCD matrixes of each microstrip TL of the T-shaped resonator and its capacitance are defined by *M*_*T*_ and *M*_*CL*_, respectively, which are equal to:

22 $$M_{T} = \left[ {\begin{array}{*{20}c} {\cos \left( {\theta_{p11} } \right)} & {jZ_{s} \sin \left( {\theta_{p11} } \right)} \\ {jY_{s} \sin \left( {\theta_{p11} } \right)} & {\cos \left( {\theta_{p11} } \right)} \\ \end{array} } \right]$$

23 $$M_{CL} = \left[ {\begin{array}{*{20}c} 1 & 0 \\ {j\omega C_{1} } & 1 \\ \end{array} } \right]$$

The ABCD matrix of the T-shaped resonator which is determined by *T*_*i*_ can be attained as:

24 $$T_{i} = M_{T} \times M_{CL} \times M_{T}$$

Therefore,

25 $$T_{i} = \left[ {\begin{array}{*{20}c} {cos^{2} \left( {\theta_{p11} } \right) - sin^{2} \left( {\theta_{p11} } \right) - 0.5\omega Z_{s} C_{1} {\text{sin}}\left( {2\theta_{p11} } \right)} & {{\text{j}}Z_{s} \sin \left( {\theta_{p11} } \right)\left( {2\cos \left( {\theta_{p11} } \right) - \omega Z_{s} C_{1} \sin \left( {\theta_{p11} } \right)} \right)} \\ {{\text{j}}y_{s} {\text{sin}}\left( {2\theta_{p11} } \right) + j\omega C_{1} cos^{2} \left( {\theta_{p11} } \right)} & {cos^{2} \left( {\theta_{p11} } \right) - sin^{2} \left( {\theta_{p11} } \right) - 0.5\omega Z_{s} C_{1} {\text{sin}}\left( {2\theta_{p11} } \right)} \\ \end{array} } \right]$$

As the second (or forth) section of the conventional λ/4 TL of the WPD depicted in Fig. 12c are replaced by the shown T-shaped resonator in Fig. 12e, their corresponding matrixes determined by (21) and (25) must be equal. This results in calculating the values of the electrical length and characteristic impedance of the microstrip TLs of the T-shaped resonators i.e., *θ*_*p11*_ and *Z*_*s*_ as follows:

26 $$\theta_{p11} = \cos^{ - 1} \left( {\sqrt {\frac{{2 + 2\cos \left( {\theta_{i} } \right) + \omega y_{c} Z_{s}^{2} C_{1} \sin \left( {\theta_{i} } \right)}}{{4 + \left( {\omega Z_{s} C_{1} } \right)^{2} }}} } \right)$$

27 $$Z_{s} = Z_{c} \sqrt {\frac{{\sin \left( {\theta_{i} } \right)}}{{\sin \left( {\theta_{i} } \right) - \omega Z_{c} C_{1} }}}$$

Obviously, as mentioned, the characteristic impedances of the employed connecting lines i.e., (*θ*_*a*_ − *θp*_*11*_), *θp*_*11*_, *θp*_*12*_ and (*θ*_*b*_ − *θp*_*11*_) in the shown proposed structure in Fig. 12d are similar and equal to (27). Furthermore, by comparing Fig. 12c and d, the first and fifth sections of the conventional λ/4 TL shown in Fig. 12c are replaced by two TLs determined by (*θ*_*a*_ − *θp*_*11*_, *Z*_*s*_) and (*θ*_*b*_ − *θp*_*11*_, *Z*_*s*_), respectively. Thus, the values of *θ*_*a*_ and *θ*_*b*_ can be calculated as

28 $$\theta_{a} = \theta_{1} + \theta_{p11}$$

29 $$\theta_{b} = \theta_{5} + \theta_{p11}$$

By comparing Fig.12c and d, it can be concluded that the third section of the conventional λ/4 TL is replaced by a modified series structure utilizing a lumped inductor, which is illustrated in Fig. 12f. The ABCD matrixes of the employed microstrip TLs and lumped inductor of the modified structure defined by *M*_*m*_ and *M*_*L*_ are as follows:

30 $$M_{m} = \left[ {\begin{array}{*{20}c} {\cos \left( {\theta_{p12} } \right)} & {jZ_{s} \sin \left( {\theta_{p12} } \right)} \\ {jY_{s} \sin \left( {\theta_{p12} } \right)} & {\cos \left( {\theta_{p12} } \right)} \\ \end{array} } \right]$$

31 $$M_{L} = \left[ {\begin{array}{*{20}c} 1 & {j\omega L_{1K} } \\ 0 & 1 \\ \end{array} } \right]$$

where *L*_*1K*_ based on the configuration of the proposed WPD depicted in Fig. 7 or Fig. 12b can be equal to either *L*_*12*_ or *L*_*13*_. Therefore, the ABCD matrix of the modified configuration illustrated in Fig. 12f can be obtained as:

32 $$T_{ML} = M_{m} \times M_{L} \times M_{m}$$

33 $$T_{ML} = \left[ {\begin{array}{*{20}c} {cos^{2} \left( {\theta_{p12} } \right) - sin^{2} \left( {\theta_{p12} } \right) - 0.5\omega y_{s} L_{1k} {\text{sin}}\left( {2\theta_{p12} } \right)} & {{\text{j}}Z_{s} \sin \left( {\theta_{p12} } \right) + j\omega L_{1k} cos^{2} \left( {\theta_{p12} } \right)} \\ {{\text{j}}y_{s} \sin \left( {2\theta_{p12} } \right) - jy_{s}^{2} \omega L_{1K} sin^{2} \left( {\theta_{p12} } \right)} & {cos^{2} \left( {\theta_{p12} } \right) - sin^{2} \left( {\theta_{p12} } \right) - 0.5\omega y_{s} L_{1k} {\text{sin}}\left( {2\theta_{p12} } \right)} \\ \end{array} } \right]$$

As the shown third section in Fig. 12c and the modified structure in Fig. 12f are equivalent, their ABCD matrixes determined by (21) and (33), respectively, must be equal. Note that, the ABCD matrix of the third section can be obtained from (21), where in this relation *i* = *3*. Thus, the value of *θ*_*p12*_ can be calculated as follows:

34 $$\theta_{p12} = \sin^{ - 1} \left( {\sqrt {\frac{{2Z_{c} \sin \left( {\theta_{i} } \right)}}{{4\omega L_{1K} + y_{s} \left( {\omega L_{1K} } \right)^{2} }}} } \right)$$

where the value of $y_{s} = Z_{s}^{ - 1}$ can be obtained from (27). Note that, in this case, to decrease the circuit size, it is necessary that 2*θp*_*12*_ < *θ*_*i*_.

**Figure 13:**
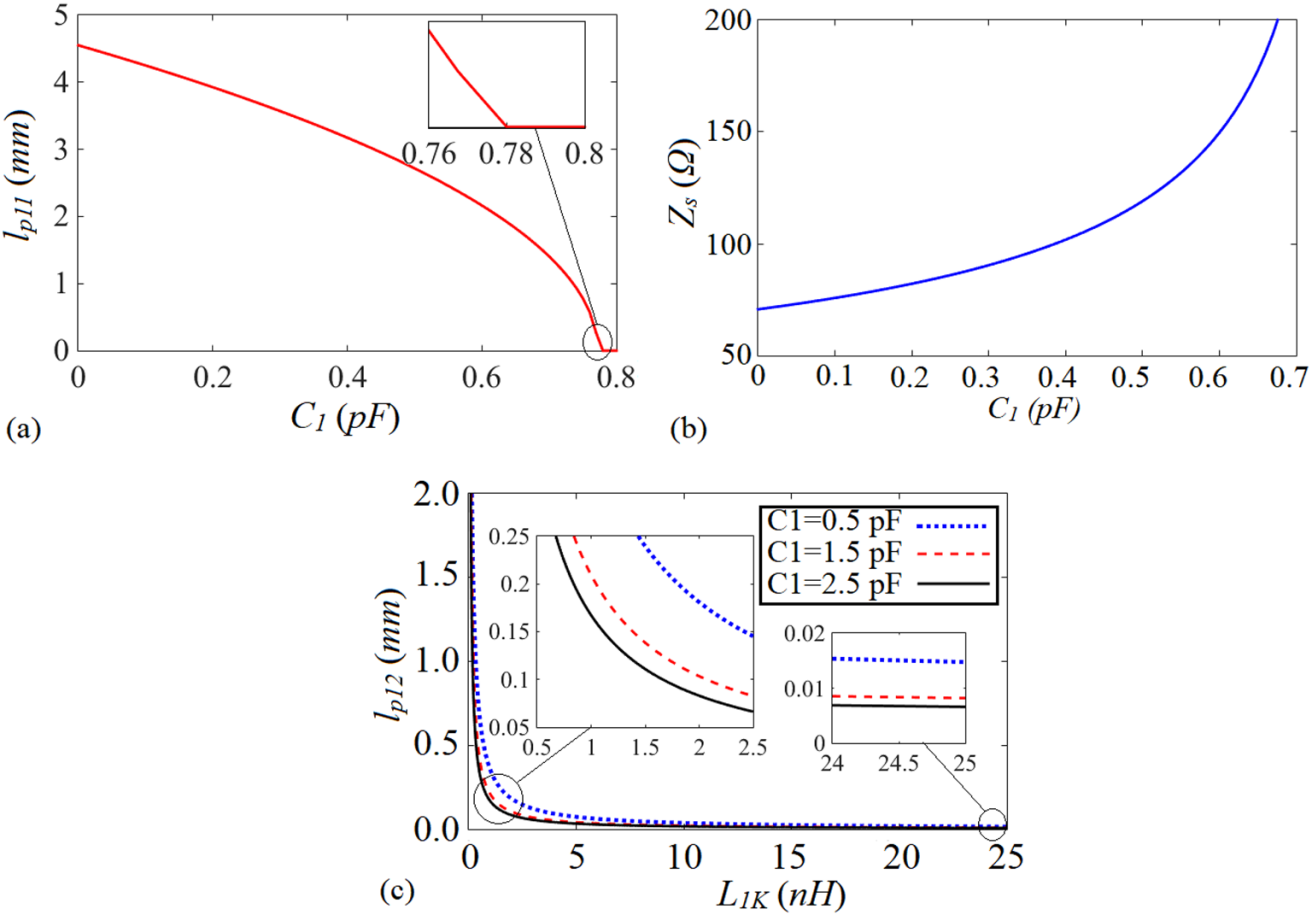
(**a**) The physical length of *l*_*p11*_ versus *C*_*1*_, (**b**) the characteristic impedance *Z*_*s*_ (*Ω*) versus *C*_*1*_ (**c**) the physical length of *l*_*p12*_ versus *L*_*1K*_*.*

Note that in (26–29) and (34), the lumped elements defined by *C*_*1*_ and *L*_*1K*_ have tunable values, which based on the desired operating frequency and the output ports power division ratio of the proposed WPD can be changed. According to (26), (27) and (34), the values of *lp*_*11*_, *lp*_*12*_ and *Z*_*s*_ versus the employed lumped elements i.e., *C*_*1*_ and *L*_*1K*_ are plotted. As can be seen from Fig. 13a, by increasing the value of *C*_*1*_, the physical length determined by *lp*_*11*_ in the T-shaped resonators decreases. When the value of the mentioned capacitance exceeds 0.78pF, the microstrip TLs determined by (*θp*_*11*_, *Z*_*s*_) in the T-shaped resonators are omitted and the connecting lines between input and output ports will be composed of the TLs defined by (*θ*_*a*_, *Z*_*s*_), (*θp*_*12*_, *Z*_*s*_) and (*θ*_*b*_, *Z*_*s*_). By enhancing the value of *C*_*1*_ to decrease the physical length *lp*_*11*_, microstrip lines with higher characteristic impedances are needed, as can be understood from Fig. 13b. According to Fig. 13c, by increasing the value of the employed inductor (*L*_*1K*_) in each of the branches of the proposed WPD, the physical length determined by *lp*_*12*_ declines significantly. As can be concluded from (34), this relation cannot be equal to zero for any value of *L*_*1K*_, and also the values of *Z*_*c*_ and *θ*_*i*_ are not equal to zero. This means that *lp*_*12*_ cannot be reduced to zero via enhancing *L*_*1K*_, which the illustrated *lp*_*12*_ versus *L*_*1K*_ in Fig. 13c proves it. Enhancing the value of *C*_*1*_ can result in declining *lp*_*12*_, but not significantly, as shown in Fig. 13c. The performed analysis confirms the results and explanations related to Fig. 6, to a great extent.

As can be seen from Figs. 7 and 12b, except for the values of the connecting inductances defined by *L*_*12*_ and *L*_*13*_, which determine the equality or inequality of the output ports power division ratio, the presented WPD is completely symmetrical around X-axis. As the values of the mentioned inductors have not been specified and determined by *L*_*1K*_, the performed analysis is independence of the equality or inequality of the power division ratio and can be applied to either the proposed equal or unequal WPD.

To validate the efficiency of the adopted technique and the performed analysis, a tunable miniaturized WPD to operate at two other operating frequencies i.e., 700 MHz and 1.2 GHz with equal and unequal power divider ratios, respectively, and capable of suppressing spurious harmonics is designed and implemented. The results of measurements of the proposed WPDs with equal and unequal output power division ratios have been discussed in the following section.

## Simulation and measurement results

**Figure 14:**
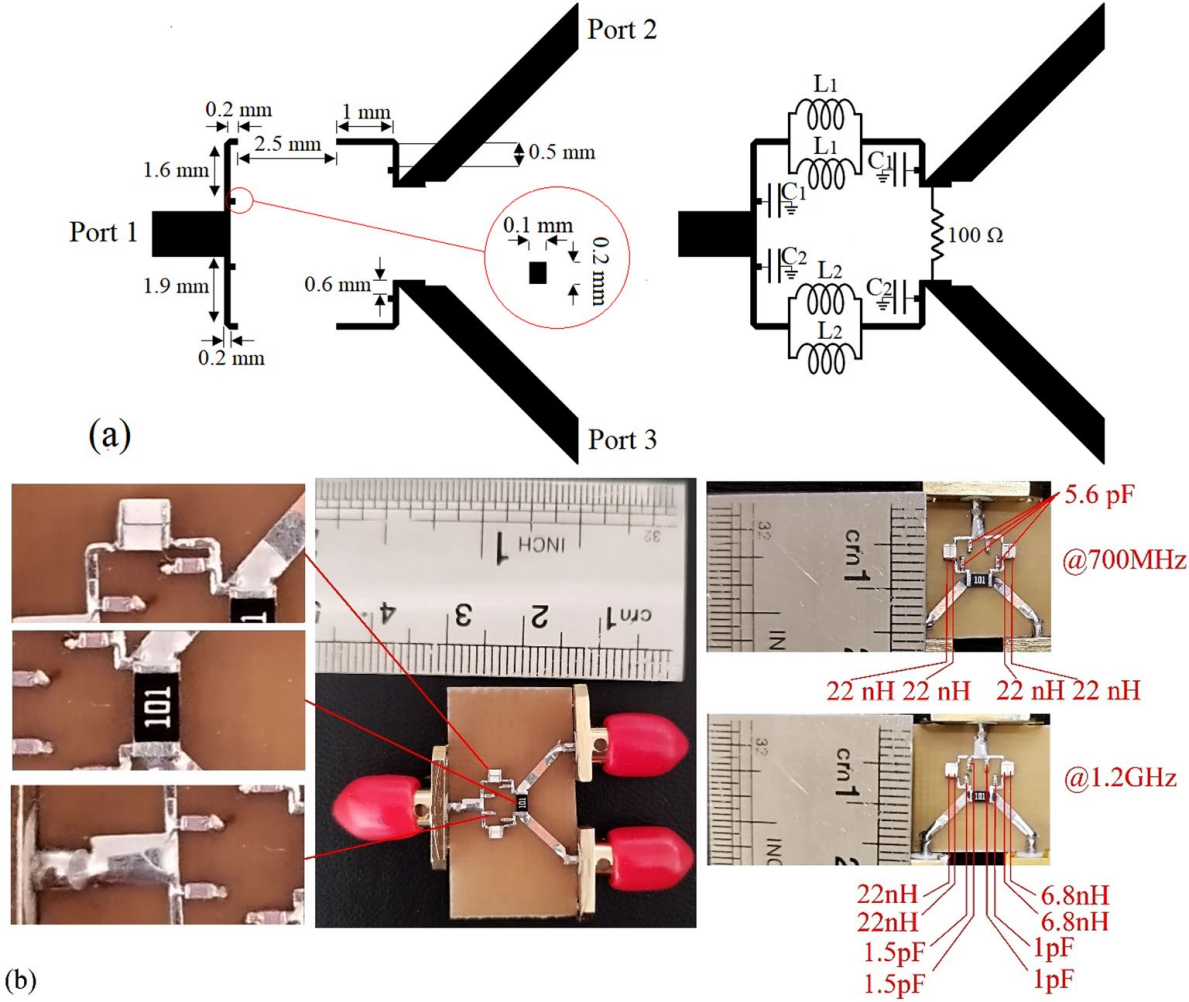
(**a**) The dimensions of both WPDs at 700 MHz and 1.2 GHz, (**b**) the implemented circuits and the photographs of the equal WPD at 700 MHz and the unequal one at 1.2 GHz.

The performed analysis has been validated via implementing a miniaturized WPD with optional equal and unequal output power division ratio. The simulations and measurements have been performed by Advanced Design System 2011 and Keysight N9917A FieldFox 18 GHz Handheld Microwave Analyzer, respectively. The proposed structure has been designed to operate at *f* = 700 MHz and *f* = 1.2 GHz with equal and unequal power division ratio, respectively. At both operating frequencies, spurious frequencies over a very wide range have been suppressed. The presented WPD has been implemented on a 1.0 mm-thickness FR4 substrate with the permittivity of 4.4 and the loss tangent of 0.0022. The dimensions of the employed microstrip TLs of the mentioned equal and unequal WPD and also the photographs of the fabricated sample at each operating frequency separately, have been depicted in Fig. 14. In the first step, a WPD with equal power division operating at 700 MHz has been designed.

**Figure 15:**
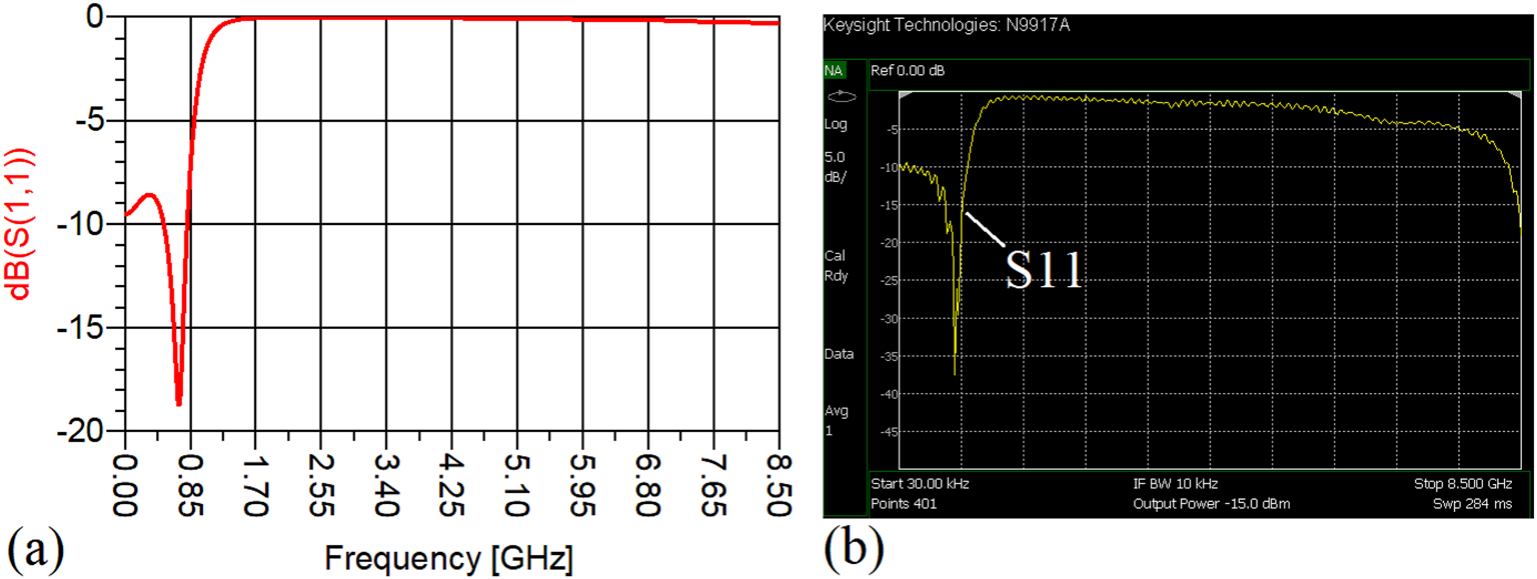
(**a**) The simulated (S11) and (**b**) the measured (S11) of the equal WPD.

**Figure 16:**
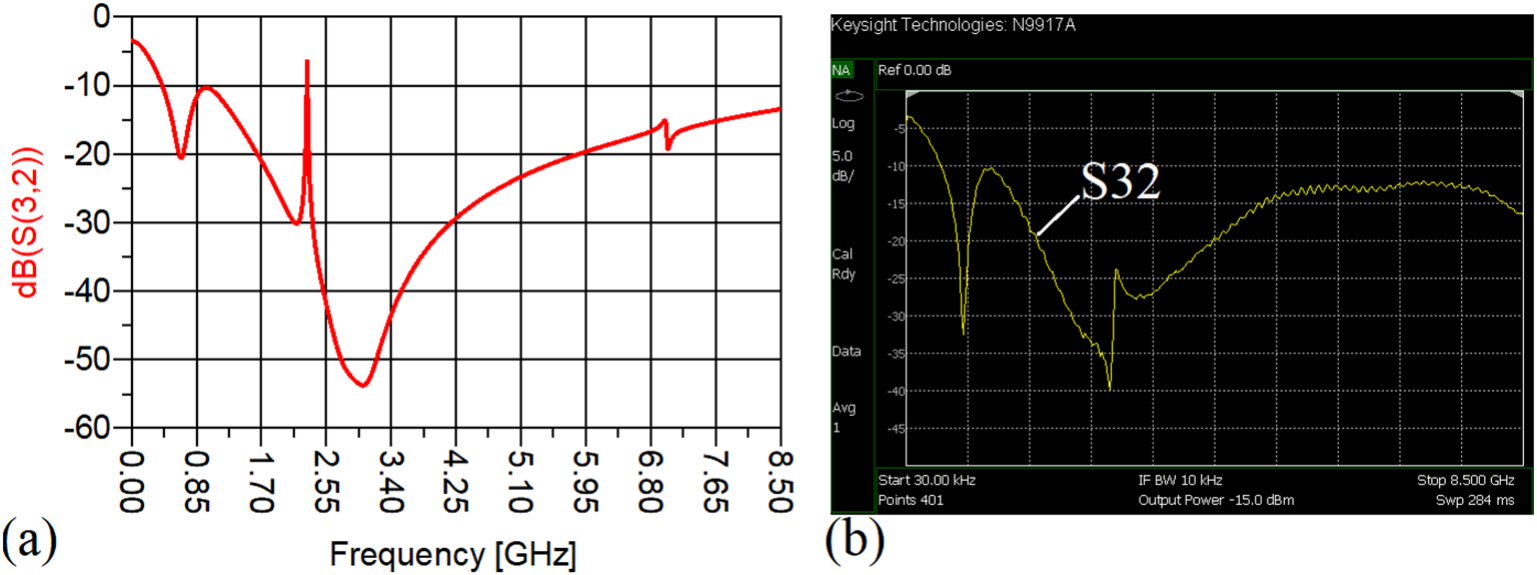
(**a**) The simulated (S23) and (**b**) the measured (S23) of the equal WPD.

**Figure 17:**
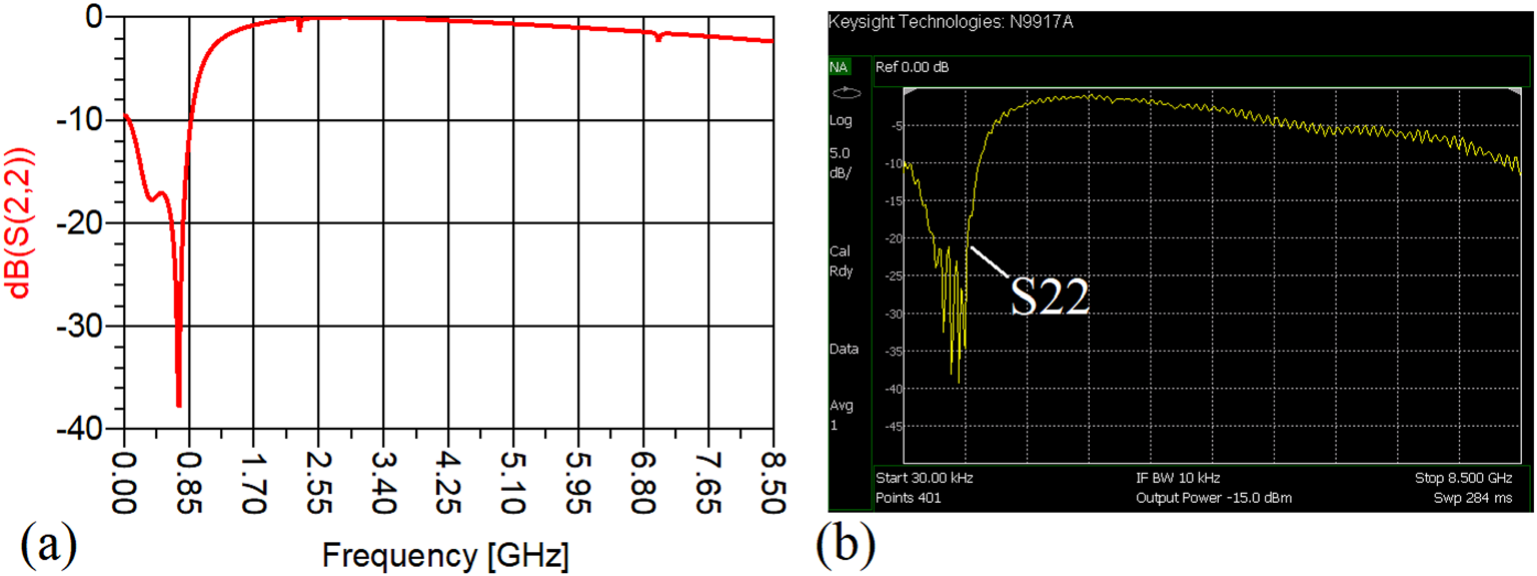
(**a**) The simulated (S22) and (**b**) the measured (S22) of the equal WPD.

**Figure 18:**
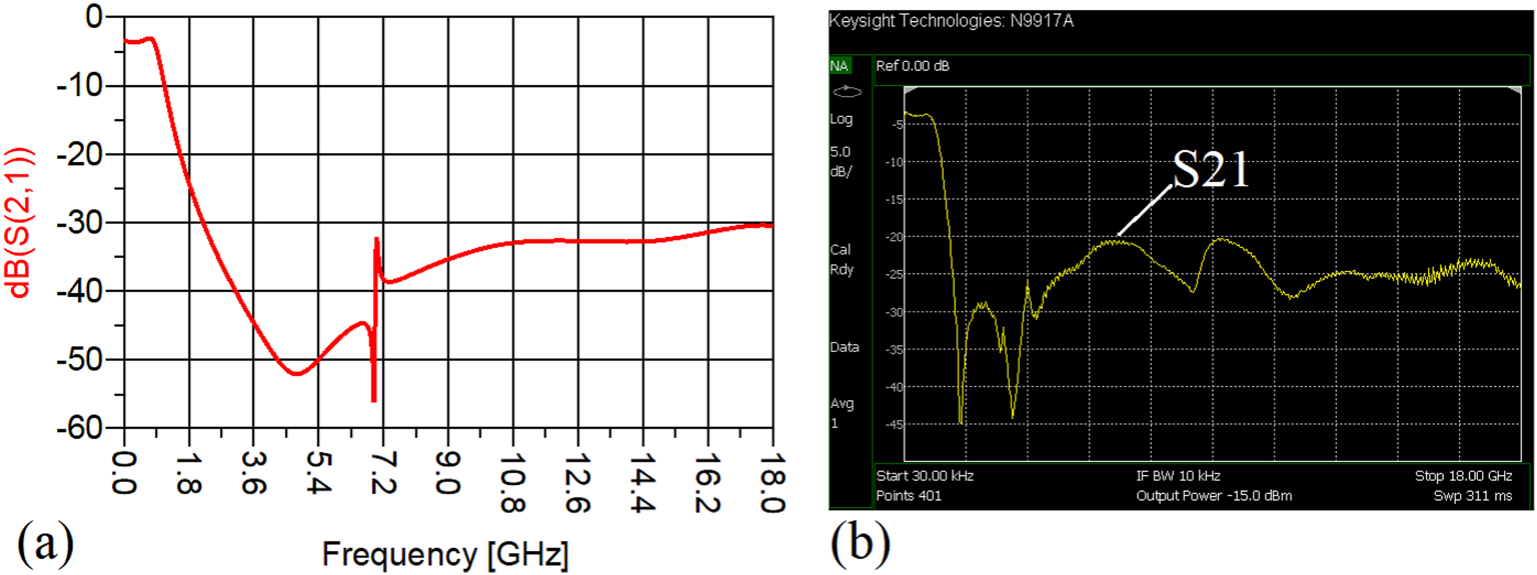
(**a**) The simulated (S21), (**b**) the measured (S21) of the equal WPD.

**Figure 19:**
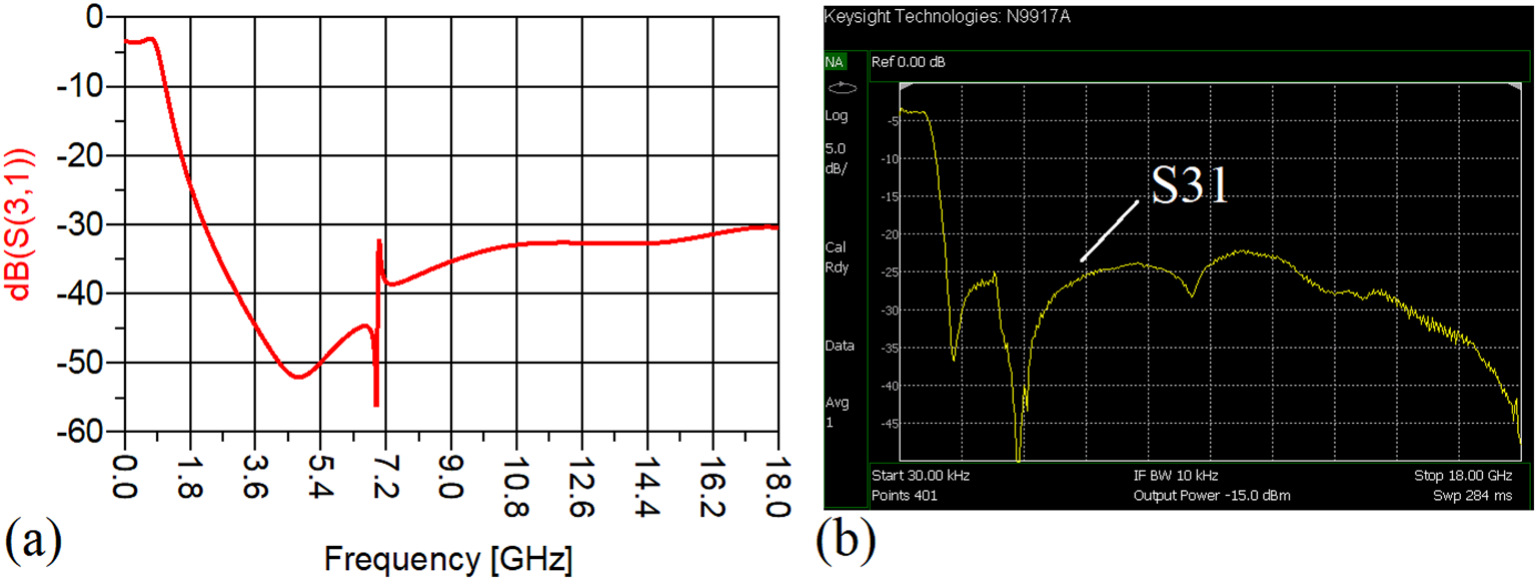
(**a**) The simulated (S31), (**b**) the measured (S31) of the equal WPD.

**Figure 20:**
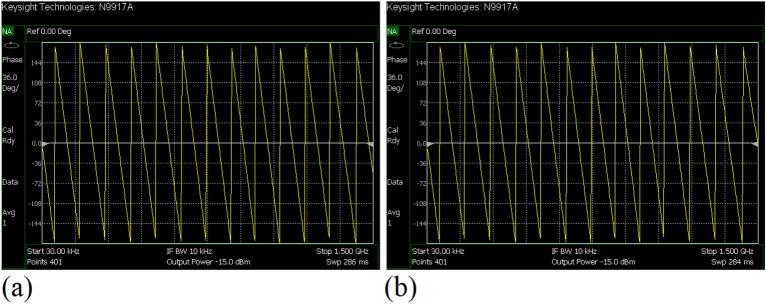
(**a**) The measured phase of (S21) and (**b**) the measured phase of (S31) of the equal WPD.

The values of the employed lumped elements are: L1 = L2 = 22 nH and C1 = 5.6 pF. The measurement and simulation results of the proposed WPD with equal power division ratio at its output ports have been shown in Figs. 15 and 20. According to Fig. 15b, the input return loss of the equal WPD is better than − 15 dB from 0.595 to 0.86 GHz. As can be seen from Fig. 16b, the measured results confirms that the isolation S23 is less than − 15 dB ranging from 0.62 to 0.935 GHz. The output return loss (S22) is better than − 15 dB from 0.25 to 0.96 GHz based on Fig. 17b. The performance of the proposed WPD with equal power division in harmonic suppression illustrated in Fig. 18b shows that the spurious frequencies over a very wide frequency band ranging from the 2nd-harmonic to 25th-harmonic have been rejected with a suppression level of better than − 22.47 dB. As can be seen in Fig. 19b, the performance of the measured S31 in harmonic suppression is to an acceptable extent similar to S21, which confirms the equal power division ratio at the output ports of the presented WPD. The output ports phases have been illustrated in Fig. 20. According to the performed measurements the phases of S21 and S31 are equal to 135.417° and 135.518°, respectively. At this operating frequency the measured scattering parameters are: S31 = − 3.48 dB, S11 = − 17.344 dB, S32 = − 21.85 dB, S22 = − 25.53 dB and S21 = − 3.467 dB. The occupied area of the proposed equal WPD is 4.1 mm × 5.8 mm, which means that compared to the conventional WPD 96.6% size reduction has been obtained at 700 MHz.

**Figure 21:**
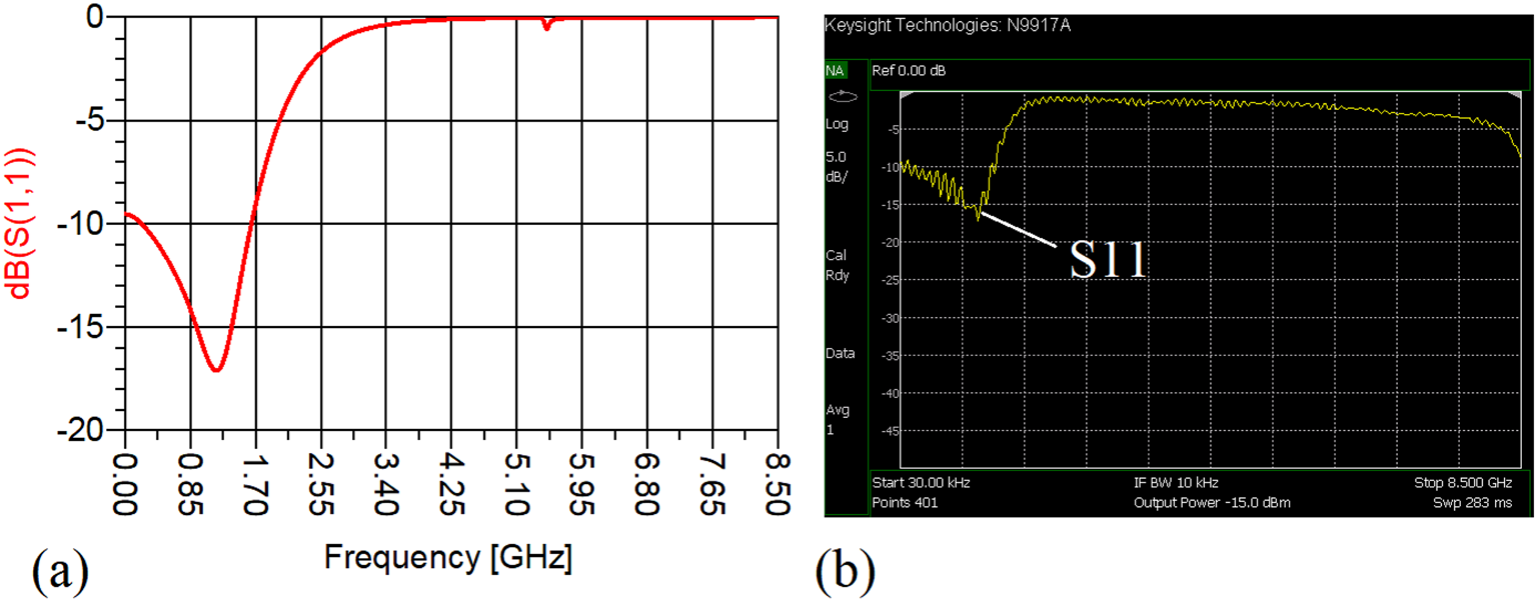
(**a**) The simulated (S11) and (**b**) the measured (S11) of the unequal WPD.

**Figure 22:**
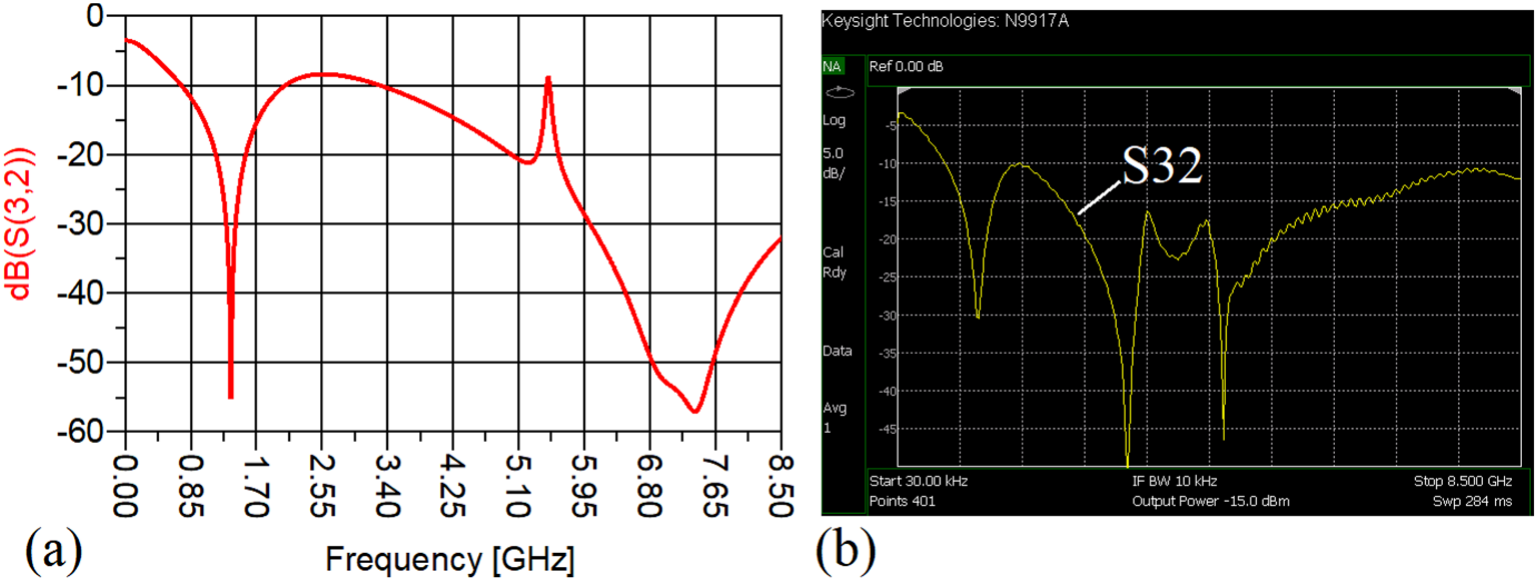
(**a**) The simulated (S23) and (**b**) the measured (S23) of the unequal WPD.

**Figure 23:**
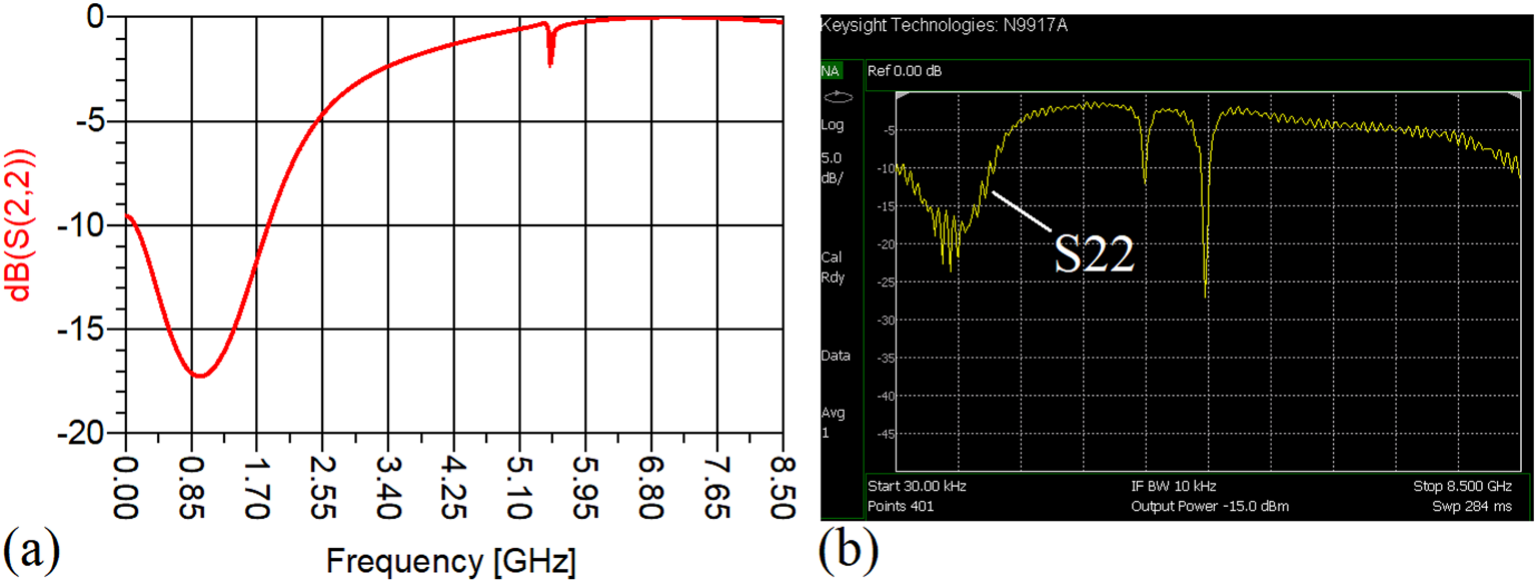
(**a**) The simulated (S22) and (**b**) the measured (S22) of the unequal WPD.

**Figure 24:**
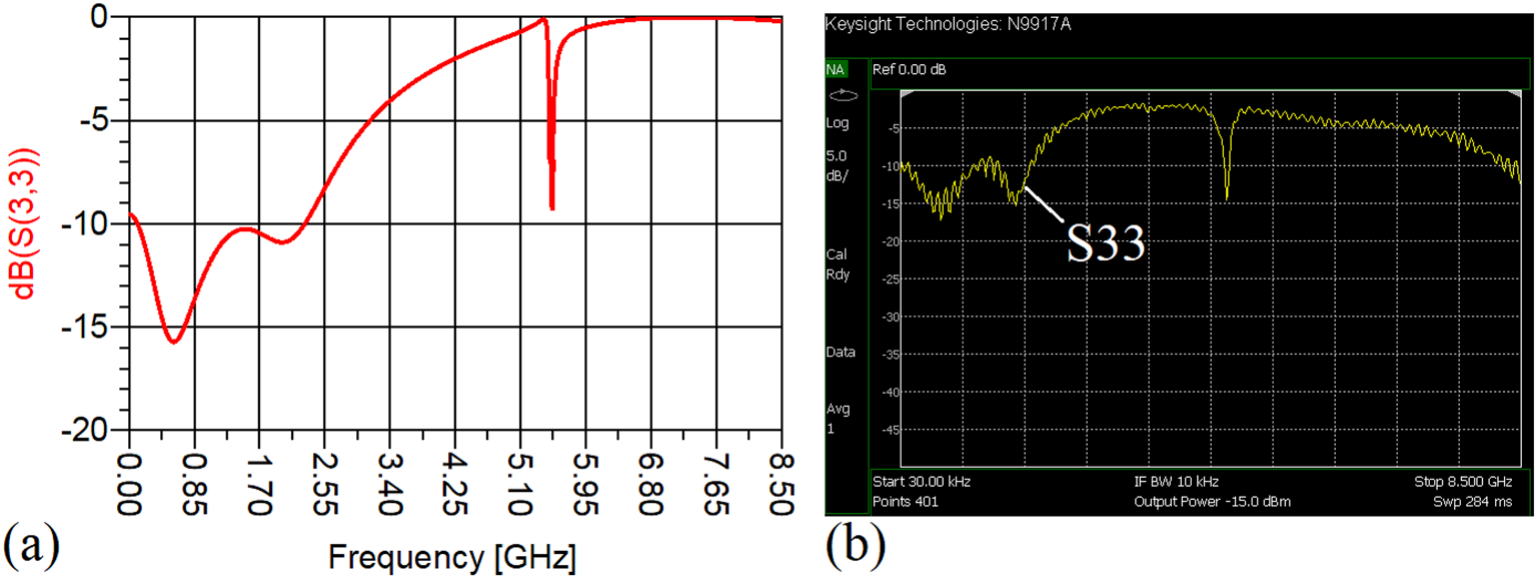
(**a**) The simulated (S33) and (**b**) the measured (S33) of the unequal WPD.

**Figure 25:**
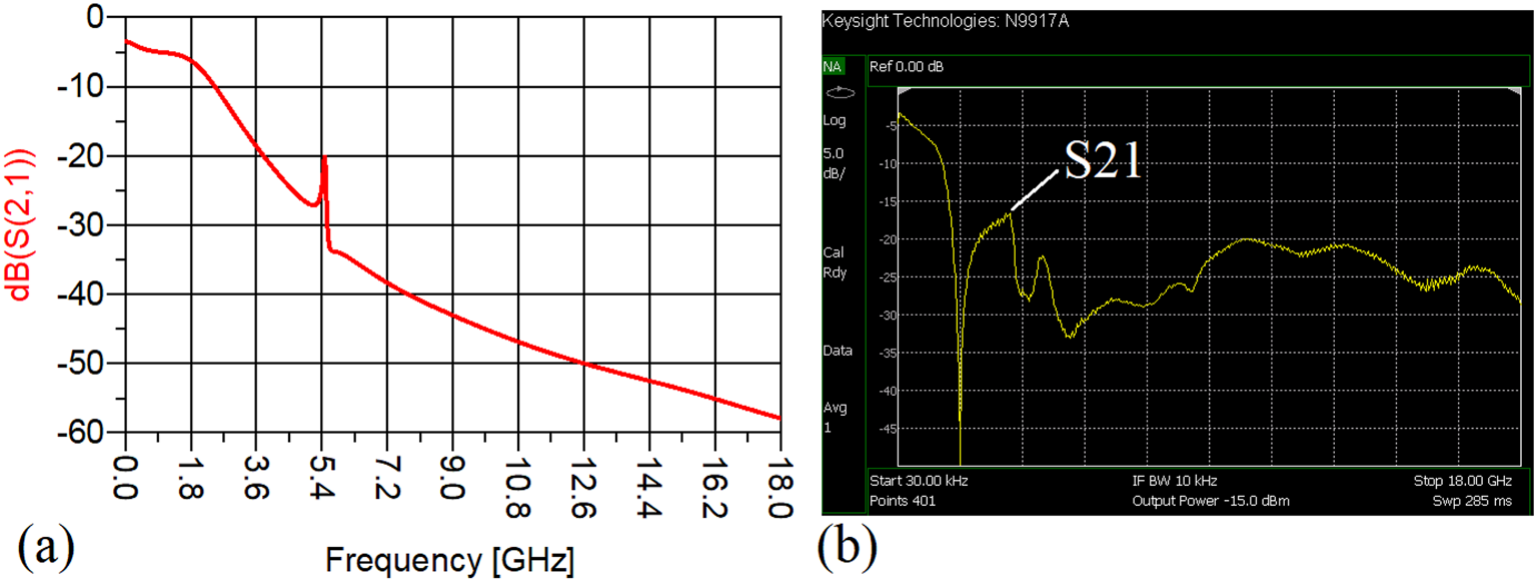
(**a**) The simulated (S21), (**b**) the measured (S21) of the unequal WPD.

**Figure 26:**
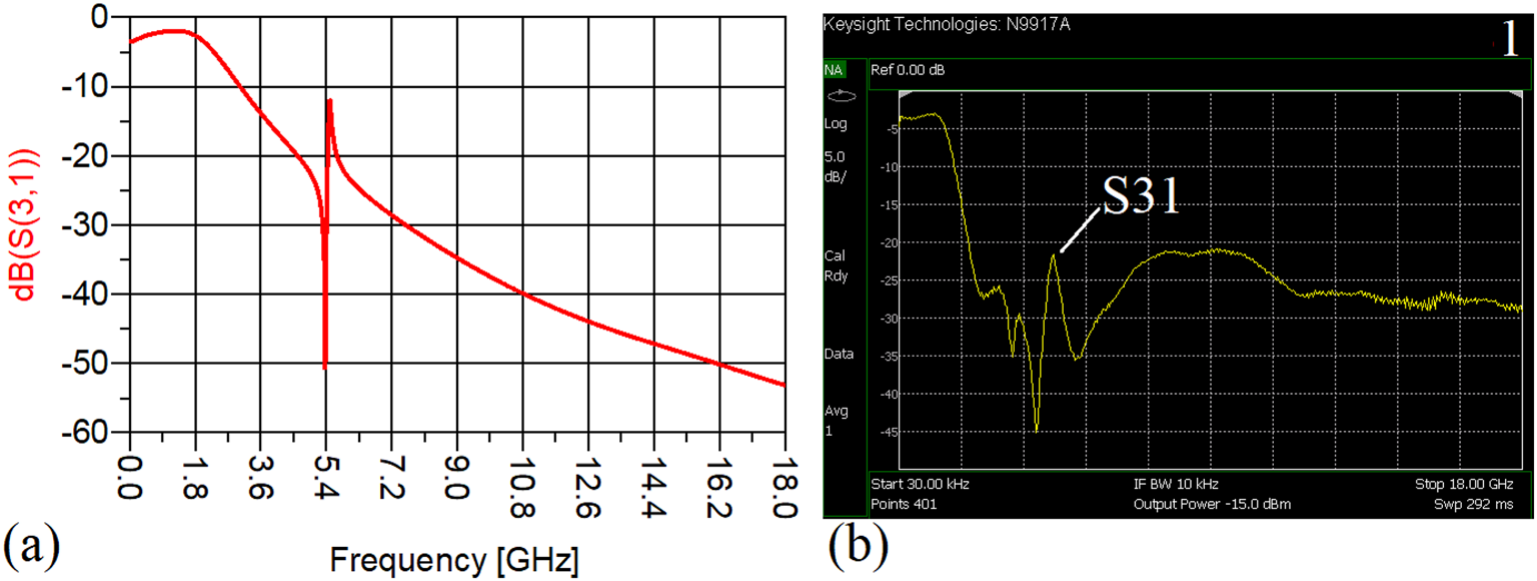
(**a**) The simulated (S31), (**b**) the measured (S31) of the unequal WPD.

**Figure 27:**
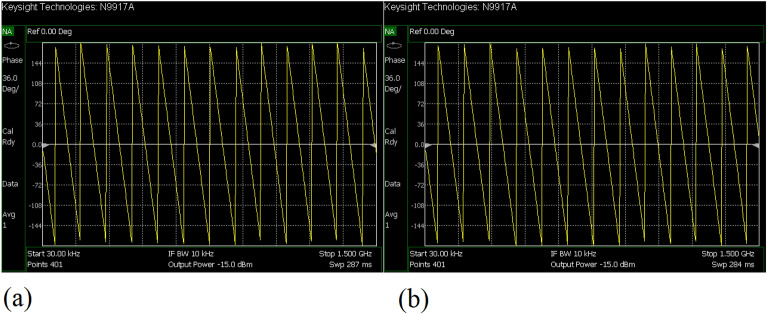
(**a**) The measured phase of (S21) and (**b**) the measured phase of (S31) of the unequal WPD.

In this stage, by changing the values of the lumped elements of the illustrated WPD in Fig. 14b with the operation frequency of 700 MHz, without varying the microstrip TLs dimensions, the unequal WPD operating at another frequency i.e., 1.2 GHz has been designed and implemented. The values of the employed lumped elements of the unequal WPD operating at 1.2 GHz are: L1 = 22 nH, L2 = 6.8 nH, C1 = 1.5 pF and C2 = 1 pF. The results of the measurements and simulations of the unequal WPD have been depicted in Figs. 21, 22, 23, 24, 25, 26 and 27. As can be observed from Fig. 21b, the input return loss (S11) of the unequal WPD is less than -15 dB from 0.87 to 1.11 GHz. On the basis of Fig. 22b, the measurement verifies that the isolation S23 is better than − 15 dB ranging from 0.765 to 1.28 GHz. As depicted in Fig. 23b, better than − 14 dB output return loss at port 2 (S22) from 0.404 to 1.23 GHz has been attained. Moreover, the output return loss at port 3 (S33) at the operating frequency of 1.2 GHz is less than − 12.2, as shown in Fig. 24b.

The performance of the proposed WPD with unequal power division in harmonic suppression, which is depicted in Figs. 25b and 26b, confirms that the spurious harmonics of S21 and S31 with a rejection factor of better than − 19.76 dB and − 21.1 dB, respectively, have been omitted. This means that over a very wide range i.e., from the 2nd-harmonic to 15th-harmonic have been suppressed. The measured phases of the output ports have been depicted in Fig. 27. According to the carried-out measurements, the phases of S21 and S31 are equal to − 17.614°and − 11.15°, respectively, which shows that there is a 6.464-degree difference between the phases of the output ports of the presented unequal WPD. The values of the measured scattering parameters at 1.2 GHz are as follows: S11 = − 15.066 dB, S32 = − 18.32 dB, S22 = − 14.01 dB, S33 = − 12.23 dB, S21 = − 8.8 dB, S31 = − 3.73 dB. The power division ratio, according to the measured S21 and S31, is 3.2:1. The circuit size of the proposed design (4.1 mm × 5.8 mm) compared to the conventional WPD with unequal power division shows that 93.5% miniaturization has been attained at 1.2 GHz. Note that, to design the unequal WPD at 1.2 GHz, the dimensions of the TLs of the previous equal divider operating at 700 MHz have not been changed; thus, the miniaturization percentage at 1.2 GHz becomes less than the obtained size reduction at 700 MHz.

The abilities of the proposed WPD are compared with several previous WPDs, which are summarized in Table 3.

**Table 3:** A comparison between the abilities of the proposed WPDs and some previous Works.

References	EPD	UPD	OEUPD	OOF	TTO/P	HS	SR
^28^	Yes	No	No	No	R&R	2nd	No
^29^	Yes	No	No	No	R&R	2nd	23%
^30^	Yes	No	No	No	R&R	2nd–8th, 10th, 17th–25th	82.8%
^31^	Yes	No	No	Yes	Varactor	No	No
^32^	Yes	No	No	Yes	Varactor	No	No
^33^	Yes	No	No	Yes	Varactor	No	No
^34^	Yes	No	No	Yes	Varactor	No	No
^35^	Yes	No	No	Yes	Varactor	No	No
^36^	No	Yes	No	No	R&R	No	75%
^37^	Yes	Yes	Yes	No	–	No	No
This work							
EPD	Yes	Yes	Yes	Yes	CLEs	2nd–25th	96.6%
UPD	Yes	Yes	Yes	Yes	CLEs	2nd–15nd	93.5%

*EPD* equal power division, *UPD* unequal power division, *OEUPD* optional equal or unequal power division, *OOF* optional operating frequency, *R&R* redesigning and reconstruction, *TTO/P* technique of tuning the operating frequency/power division ratio, *CLEs* changing lumped elements, *HS* harmonic suppression, *SR* size reduction.

The scattering parameters of the proposed WPDs and some relevant published designs have been compared in Table 4.

**Table 4:** A comparison between the frequency response of the proposed WPDs and some previous Works.

References	^28^	^29^	^30^	^31^	^32^	^33^	^34^	^35^	^36^	^37^		This work	
										EPD	UPD	EPD	UPD
Insertion loss (dB)	3.85	NA	3.3	< 4.30	< 5.40	< 3.5	< 4.50	< 4.56	1.9/4.5	4.14/4.5	3.3/6.8	3.46/3.48	3.73/8.8
Return loss (dB)	> 20	> 15	> 14	> 20	> 15	> 20	> 20	> 20	> 20	> 12.5	> 10	17.34	15.066
Isolation (dB)	20	> 13	15	> 20	> 16	> 20	> 25	> 12	> 20	> 10	> 11	21.85	18.32

As can be seen from Tables 3 and 4, the proposed structure has brought about considerable features such as optional operating frequency and power division ratio, simple structure, harmonic suppression, size reduction and also acceptable frequency response.

Finally, the implemented WPDs can be used in reconfigurable radio systems, for example, RF self-interference cancellation system^41^.

## Conclusion

In this paper, a modified π-shaped resonator, which is a combination of microstrip TLs and lumped elements, has been used instead of the quarter-wavelength TL of the conventional WPD. Adopting this modified resonator has resulted in designing a compact divider which its operating frequency and output ports power division ratio can be controlled and selected optionally via changing the values of its lumped elements without manipulating the dimensions of the utilized microstrip lines. Moreover, by employing the mentioned resonance cell not only the occupied area of the designed WPD at each desired operating frequency has been decreased considerably, but also the spurious harmonics over a very wide range have been suppressed. On the basis of the performed analysis, a WPD which not only its operating frequency can be changed to work at 0.5, 1.0, 1.5 and 2 GHz, but also its power division equality or inequality can be selected optionally at each of the mentioned frequencies, has been designed and simulated. Then, to validate the obtained theoretical and simulation results, a WPD capable of operating at 700 MHz and 1.2 GHz optionally with equal and unequal power division ratios, respectively, has been designed and implemented. At the first and second operating frequencies, the spurious harmonics from the 2nd to 25th and the 2nd to 15th, respectively, have been suppressed. Moreover, almost 96% and 93% size reduction at 700 MHz and 1.2 GHz, respectively, have been achieved. The power at the output ports of the WPD at 1.2 GHz has been divided unequally as S21 = − 8.8 dB and S31 = − 3.73 dB, which proves that the inequality ratio is 3.2:1.

## Data Availability

The calculated results during the current study are available from the corresponding author on reasonable request.
